# Spinopelvic alignment and low back pain after total hip arthroplasty: a scoping review

**DOI:** 10.1186/s12891-022-05154-7

**Published:** 2022-03-15

**Authors:** Mohammadreza Pourahmadi, Mohammad Sahebalam, Jan Dommerholt, Somayeh Delavari, Mohammad Ali Mohseni-Bandpei, Abbasali Keshtkar, César Fernández-de-Las-Peñas, Mohammad Ali Mansournia

**Affiliations:** 1 Rehabilitation Research Center, Department of Physiotherapy, School of Rehabilitation Sciences, Iran University of Medical Sciences, Tehran, Iran; 2 Bethesda Physiocare, Bethesda, MD USA; 3 Myopain Seminars, Bethesda, MD USA; 4 Department of Physical Therapy and Rehabilitation Science, School of Medicine, University of Maryland, Baltimore, MD USA; 5 Center for Educational Research in Medical Sciences (CERMS), Department of Medical Education, School of Medicine, Iran University of Medical Sciences, Tehran, Iran; 6 Pediatric Neurorehabilitation Research Center, University of Social Welfare and Rehabilitation Sciences, Tehran, Iran; 7 University Institute of Physical Therapy, Faculty of Allied Health Sciences, University of Lahore, Lahore, Pakistan; 8 Department of Health Sciences Education Development, School of Public Health, Tehran University of Medical Sciences, Tehran, Iran; 9 Department of Physical Therapy, Occupational Therapy, Rehabilitation and Physical Medicine, Universidad Rey Juan Carlos, Alcorcón, Madrid, Spain; 10 Cátedra de Investigación y Docencia en Fisioterapia: Terapia Manual y Punción Seca, Universidad Rey Juan Carlos, Alcorcón, Madrid, Spain; 11 Department of Epidemiology and Biostatistics, School of Public Health, Tehran University of Medical Sciences, Poursina St., Shanzdah-e Azar St., P. O. Box: 6446-14155, Tehran, Iran

**Keywords:** Arthroplasty, Replacement, Hip, Spinopelvic alignment, Spine, Low back pain, Review

## Abstract

**Objective:**

Spinopelvic alignment is increasingly considered as an essential factor for maintaining an energy-efficient posture in individuals with normal or pathological status. Although several previous studies have shown that changes in the sagittal spinopelvic alignment may occur in patients undergoing total hip arthroplasty (THA), no review of this area has been completed so far. Thus, the objective of this scoping review was to summarize the evidence investigating changes in spinopelvic alignment and low back pain (LBP) following THA.

**Data sources:**

We adhered to the established methodology for scoping reviews. Four electronic databases were systematically searched from inception-December 31, 2021.

**Study selection:**

We selected prospective or retrospective observational or intervention studies that included patients with THA.

**Data extraction:**

Data extraction and levels of evidence were independently performed using standardized checklists.

**Data synthesis:**

A total of 45 papers were included in this scoping review, involving 5185 participants with THA. Pelvic tilt was the most common parameter measured in the eligible studies (*n* = 26). The results were not consistent across all studies; however, it was demonstrated that the distribution of pelvic tilt following THA had a range of 25° posterior to 20° anterior. Moreover, decreased sacral slope and lower pelvic incidence were associated with increased risk of dislocation in patients with THA. Lumbar spine scoliosis did not change significantly after THA in patients with bilateral hip osteoarthritis (5.50°(1.16°) vs. 3.73°(1.16°); *P*-value = 0.29). Finally, one study indicated that LBP improvement was not correlated with postoperative changes in spinopelvic alignment parameters. Several methodological issues were addressed in this study, including no sample size calculation and no type-I error adjustment for outcome multiplicity.

**Conclusions:**

Changes in spinopelvic alignment may occur after THA and may improve with time. Patients with a THA dislocation usually show abnormal spinopelvic alignment compared to patients without a THA dislocation. LBP usually improves markedly over time following THA.

**Supplementary Information:**

The online version contains supplementary material available at 10.1186/s12891-022-05154-7.

## Background

Assessment of spinopelvic alignment is gaining increasing importance and attention, not only in spinal surgery but also in hip surgery [1]. Spinopelvic alignment is a complex chain of correlations from the spine to the pelvis and that changes in one region of the spine can result in reciprocal changes in other spinopelvic regions with potential alignment consequences [2]. The literature has shown that imbalanced spinopelvic alignment is associated with worse function and poor quality of life in patients with spine and hip disorders [3]. Hence, efficient performance of daily human activities requires an ideal coordinated motion between the spine, pelvis, and hips. A good example of normal relative motion between the adjacent segments is stand-to-sit movement, in which flattening (or flexion) of the lumbar spine, a posterior tilt of the pelvis, and flexion of the hips happen [4]. Spinopelvic alignment and kinematic imbalance following total hip arthroplasty (THA) may change the functional position of the acetabulum, creating a potential for dislocation [5, 6].

THA is one of the most common, cost-effective, and clinically successful surgeries performed today for the treatment of arthritic hip disorders [7–9]. It provides reliable outcomes for patients suffering from end-stage degenerative hip osteoarthritis (OA), especially pain relief, functional restoration, and overall improved quality of life [8]. Although THA is referred to as ‘*the operation of the century*’ in 2007 [10], it not without potential complications and consequences. Healy et al. (2016) [9] listed the potential complications of THA including bleeding, wound complication, thromboembolic disease, neural deficit, periprosthetic fracture, dislocation or instability, abductor muscle disruption, deep periprosthetic joint infection, vascular injury, implant loosening, osteolysis, and so forth. Furthermore, changes in the spinopelvic alignment may occur in patients after THA [11]. Heckmann et al. (2018) [6] alleged that spinopelvic imbalance may serve as a causative factor for late dislocations after THA. Lateral spine-pelvis-hip radiographs may provide a radiographic explanation for both instability and the direction of the dislocation. Various spinopelvic alignment parameters have been evaluated before or after THA, including pelvic incidence (PI), pelvic tilt (PT), sacral slope (SS), sagittal vertical axis (SVA), lumbar lordotic (LL) angle, thoracic kyphosis (TK) angle, and coronal lumbar angles [6, 11–13]. The PI, which is the algebraic sum of the PT and SS, is a constant morphologic parameter that helps clinicians to predict the physiologic individual sagittal range of motion of the pelvis [14]. The PI does not change with different ages and in between the sexes [15], and it is an important parameter for determining the spinal balance [16]. The PT, SS, and LL angle are functional parameters with body position-dependent values [14]. Available literature suggests that abnormal spinal alignment may be associated with LBP [17]. It is also reported that the prevalence of LBP among patients undergoing THA varies between 21.2 and 60.4% [18].

Due to the importance of spinopelvic alignment and kinematics following THA, this scoping review was conducted to identify and summarize the existing literature and indicate methodological gaps in the available body of knowledge before providing a framework for future research. Scoping reviews are a form of knowledge synthesis that address an exploratory research question, often aiming to approach complicated, broad, or fragmented areas of research that have not received much attention in the literature [19]. Since there are no published systematic reviews on this topic, this scoping review aims to provide a comprehensive overview of spinopelvic alignments and low back pain (LBP) following THA. In this scoping review we did not assess the relationship between spinopelvic alignment and LBP following THA.

## Methods

One reviewer (M.P.) conducted an exhaustive scoping search once the authors decided on the aim of the review. Thus, authors ensured that the objective had not been addressed by previous reviews. This scoping review used the framework proposed by Arksey and O’Malley (2005) [20], with consideration given to suggestions from Levac et al. (2010) [21] to guide the methodology. A scoping review does not require ethical approval and patient consent since it does not include any new data collection. Because PROSPERO does not currently accept protocols for scoping reviews, a review protocol was not registered in any registry. We reported following the Preferred Reporting Items for Systematic Reviews and Meta-Analyses extension for Scoping Reviews Checklist [22]. Five steps were followed:

i) Identify the research question
ii) Identify relevant studies
iii) Study selection
iv) Chart the data
v) Collate, summarize, and report the results

### Identify the research question

Our research questions were as follows:

- What are the volume, yearly distribution, spinopelvic alignment parameters, and LBP measured in previously published studies in the field of THA?
- What critical areas of methodological improvement are needed to optimize the quality of future studies?

To formulate a search strategy for the current scoping review, the PI/ECOT(S) method was employed, as described by Sackett et al. (2000) [23].

**P** (Population)—participants of all ages who had undergone THA (10th revision, 2020 ICD-10-CM diagnosis code Z96.64). In the present study, THA was defined as surgery in which the diseased ball and socket of the hip joint are completely removed and replaced with artificial materials. No restriction for the reason of THA was imposed. No studies were included in which only surface replacement arthroplasty (SRA) of the hip was performed.

**I/E** (Intervention/Exposure)—a THA procedure with no limitation on approach performed (i.e.*,* anterior, posterior, or lateral) and materials and implants being used.

**C** (Comparator)—any comparator; no comparator.

**O** (Outcomes)—the outcomes of this scoping review were LBP and spinopelvic parameters. Spinopelvic parameters extracted in this scoping review are defined in Table 1.

**Table 1:** Spinopelvic alignment parameters definitions and their normative ranges/values

Spinopelvic parameter	Definition	Normative range/values
Cervical lordosis (CL)	The angle between the lines tangent to the posterior aspect of C_2_ and C_7_ vertebral bodies [80].	20° − 35° [81]
Thoracic kyphosis (TK)	The angle between the superior endplate of T_5_ and the superior endplate of T_12_ [16].	20° − 45° [82]
Lumbar lordosis (LL)	The angle between the superior endplate of L_1_ and the superior endplate of S_1_ [16].	So wide (30° − 80° using the Cobb method) [83]
Lumbar scoliosis	A lateral curvature of the lumbar spine with torsion of the spine and a disturbance of the sagittal profile [84]. Lumbar scoliosis is measured using the Cobb angle, which is the angle between the two most tilted vertebrae of a given scoliotic curve as measured on a coronal radiograph [85].	Cobb angle > 10° in skeletally mature patients [86]
T_1_ spinopelvic inclination (T_1_Spi)	The angle between the line drawn from the centroid of T_1_ and the center of the bicoxofemoral axis and the vertical plumb line [87].	−13° − + 5° (average = − 4.67°) [88]
Pelvic tilt (PT)	The angle between the vertical line and line joining the middle of sacral endplate to the center of the bicoxofemoral axis [89].	13° (6°) [90]
T_1_ pelvic angle (TPA)	The angle between the line from the femoral head axis to the centroid of T_1_ and the line from the femoral head axis to the middle of the S_1_ endplate [88].	−6° − + 25° (average = 8.28°) [88]
Sacral slope (SS)	The angle between the superior endplate of S_1_ and a horizontal reference on sagittal imaging of the lumbosacral spine [89].	Approximately 33° − 49° (average = 41°) [90]
Pelvic incidence (PI)	The angle between the line perpendicular to the sacral endplate at its midpoint and the line connecting this point to the axis of the femoral heads [89]. Pelvic incidence = sacral slope + pelvic tilt [56].	Approximately 45° − 65° (average = 55°) [90]
Pelvic inclination angle (PIA)	The angle between the line connecting the anterior boarder of the sacral promontory with the upper border of the symphysis and a horizontal line [91].	60° [91]
Anterior pelvic plane angle (APP)	The angle between the vertical line and the line connecting the pubic symphysis and the bilateral anterior superior iliac spine midpoint (anterior pelvic plane) [16, 57].	−5° − + 5° [16]
Sagittal vertical axis (SVA)	Distance between the C_7_ plumb line and the postero-superior edge of S_1_ [92].	< 30 mm [93]
Spinosacral angle (SSA)	The SSA angle is defined by the angle connecting the center of the C_7_ vertebra to the center of the S1 endplate and the line parallel to the superior S_1_ endplate [94].	135° (8°) [94]
Cup/ acetabular (lateral) inclination (CI)	The angle between the transverse axis and the articular side of the acetabular cup. Measurement of this angle can be done by drawing a line through the medial and lateral margins of the cup and measuring the angle with the transischial tuberosity line [95].	30° − 50° [75]
Functional cup (acetabular) inclination (FI)	The angle between the pelvic longitudinal axis and the acetabular axis when this is projected onto the coronal plane [29].	43.7° − 55.9° [96]
Sagittal plane cup anteversion (CA)	The angle between the line tangent to the anterior and posterior edges of the acetabulum and the horizontal plane [97]. In the transverse plane, it is the angle formed by the line tangent to the anterior and posterior edges of the acetabulum and the sagittal axis [97].	5° − 25° [30]
Functional cup (acetabular) anteversion (FCA)	The angle can be calculated using the Lewinnek’s formula: cup anteversion angle = arc sin (D_1_/D_2_). D_1_ is the distance of the short axis of an ellipse drawn perpendicular to the long axis of the acetabular component and D_2_ is the distance of the long axis [31].	It depends on several variables such as planned inclination, planned anteversion, standing pelvic tilt, and sitting pelvic tilt [98]

**T** (Time)—all studies were considered; studies were not limited according to time of follow-up.

**S** (Study design)—Studies with the design of clinical trials and observational (i.e.*,* cohort, cross-sectional, and case-control) were considered eligible. Articles published only as conference proceedings/abstracts, narrative reviews, systematic reviews, news/magazine articles, case reports, or where only published in protocol stage were excluded.

### Identifying relevant studies

#### Licensed journal databases

One author (M.P.) systematically searched for peer-reviewed articles, from inception to December 31, 2021, in the following licensed databases: PubMed/MEDLINE, Scopus, Embase®, and the Cochrane Central Register of Controlled Trials (CENTRAL). Electronic search strategies were constructed based on the combined keywords: *hip, arthroplasty, low back pain, spinopelvic alignment,* and *spinopelvic range of motion* to identify human studies that measured spinopelvic alignment and ROM following THA. A combination of Medical Subject Headings (MeSH; MEDLINE), Emtree medical (Embase®) terms, and free text words in research equations with ‘OR’ and ‘AND’ Boolean operators were used. Free text words were selected from the indexed keywords of most relevant original studies and reviews (e.g., [24–27]) in Scopus. Free text words were also selected from the synonyms of all keywords used in the text of relevant studies. Search terms around the types of study were not used to prevent limiting the search. The search strategy was initially created in PubMed/MEDLINE (NLM) and then translated into the other databases. Details of PubMed/MEDLINE (NLM) database and other databases search syntaxes are presented in Supplement file 1. We did not review content from file sources that were from mainstream publishers (e.g., Sage, Wiley, ScienceDirect, Springer, Taylor & Francis, and BMJ), as we expected these to be captured in our broader search strategy. Our searches had no language restrictions.

#### Grey literature

We searched for ongoing and unpublished studies in the Register for Clinical Trials (https://clinicaltrials.gov/) and the WHO’s International Clinical Trials Registry Platform (https://www.who.int/ictrp/en/).

#### Hand searching

Citation tracking in Scopus and reference list scanning of the selected studies and relevant reviews were checked for eligible studies to ensure comprehensiveness. Additionally, the table of contents of the *Journal of Arthroplasty* and the *Journal of Bone & Joint Surgery— American Volume* was reviewed.

### Study selection

After completion of all database searches, the citations were imported into the EndNote reference management software (version X9.1; Clarivate Analytics Inc., Philadelphia, PA, USA), where duplicate citations were removed automatically and double-checked manually. Articles were assessed for inclusion through a two-stage process. The first stage screening was of titles and abstracts and was done independently by two reviewers (M.P. and M.S.). Any disputes were discussed until a consensus was reached between the reviewers. If consensus was not possible, a final decision was made by a third expert reviewer (A.K.). If a study met all of the criteria, then the full text of the study was assessed for eligibility. Furthermore, a full-text review was undertaken if the title and abstract did not provide adequate information. A table named “list of excluded articles with reasons” was also established for the excluded studies.

### Chart the data

#### Data extraction

Data abstraction from the selected articles was carried out by two reviewers (M.P. and M.S.), as suggested by Arksey and O’Malley (2005) [20]. An electronic spreadsheet (Microsoft Excel, Microsoft Office 365, Redmond, WA, USA) was designed according to the objectives specified in this scoping review, and the following data were extracted: study design, number of included participants, objective(s) of the study, arthroplasty approach, outcomes of the study, key finding(s), and any other relevant details. The data extraction tool was piloted with three articles of varied methodological approaches in order to ensure it would collect the correct and effective information. This process was verified by one researcher (M.P.). It was an iterative process in which there were many changes during each round. Any disagreements were resolved by consensus. Following the completion of the data extraction process, one author (M.P.) double-checked the extracted data as a quality control. As customary with scoping reviews, eligible studies were not formally assessed for risk of bias. However, the Oxford Centre for Evidence-Based Medicine (CEBM) grade of evidence was used for the assessment of each included study. Levels of evidence ranged from one to five, with one indicating the highest quality of evidence and five indicating the lowest quality of evidence (eTable 1).

### Collating, summarize, and report the results

This study employed a *‘descriptive-analytical’* method within the narrative tradition to summarize the data and include the following:

- Descriptive numerical analysis: The nature and distribution of the included articles were assessed concerning the total number of articles, year of publication, country of origin, study population, study design, and journals where the articles were published.
- Narrative summary of included study findings: We reported the studies’ results according to the present study outcomes. Where relevant and where possible, we extracted the point estimates and 95% confidence intervals/ standard deviations provided.
- Implication of results: We reported the findings according to our objective of describing the published literature on spinopelvic alignment changes after THA. Furthermore, we identified the gaps in the current literature base.

## Results

### Papers designs and participants

**Fig. 1:**
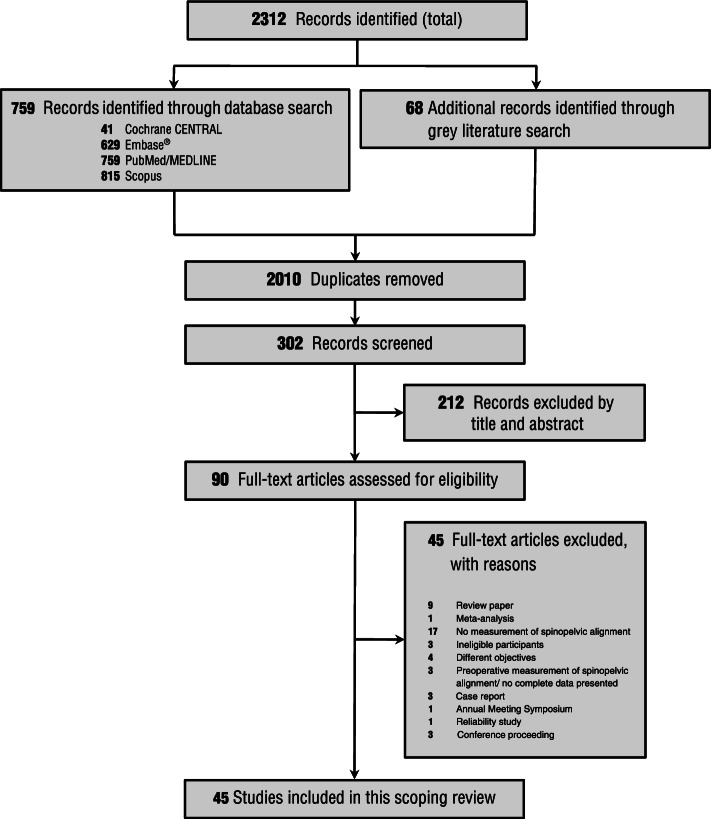
Scoping Review Inclusion Flow Diagram

From 2312 identified records, we selected 90 full-text reports (Fig. 1). Forty-five papers were excluded for the reasons summarized in Fig. 1 and eTable 2. Overall, data were extracted from 45 papers (Fig. 1). About 78% of the included papers were conducted in the United States and Japan. The Journal of Arthroplasty published around 22% of the included papers (eTable 3). Forty-four out of 45 studies (~ 98%) were published in English, and one paper was published in Japanese [28]. The original studies sample sizes varied from 20 to 509 with a median [interquartile range; IQR] of 84 [50–138]. Most papers included participants ≥60 years of age. Lateral and anterolateral approach surgeries for THA were commonly performed [16, 17, 29–42]. Reassessments using radiographic images were performed between 0 to 120 months after THA with a median and IQR of 12 and 6–24, respectively. Details of the references are provided in Additional file 2. Additionally, A review of grey literature identified several relevant records in this area (eTable 4). According to Oxford Centre for Evidence-Based Medicine criteria, 21 papers met Level 2 level of evidence [6, 16, 32, 33, 35, 37, 41–55], while 24 papers met Level 3 due to retrospective nature and quality of the study [3, 11, 17, 28–31, 34, 36, 38–40, 56–67].

### Spinopelvic alignments and LBP

PT was the most commonly parameter measured by the included studies (*n* = 26), followed by SS (*n* = 25), PI (*n* = 20), LL (*n* = 19), cup (acetabular) anteversion (CA; *n* = 14), and cup (acetabular) inclination (CI; *n* = 12) (Table 2). Pelvic motion were measured in only one study [47]. Moreover, LBP was measured in eight studies [11, 17, 32, 39, 41, 53, 55, 65]. The results revealed that there is controversy about the spinopelvic alignment parameters in patients with THA. Carender et al. (2020) [66] reported that the prevalence of decreased spinopelvic motion was 34.2% in 228 patients who underwent primary THA. Three studies showed that the distribution of PT following THA had a range of 25° posterior to 20° anterior [35, 49, 60]. In a study conducted by Kanto et al. (2019) [16], changes in PT were observed postoperatively in 59% of included patients. Furthermore, some studies reported that patients with high degrees of posterior PT showed an increased posterior PT 5–10 years after THA [35, 60]. Fixed posterior PT during standing, lower LL, and decreased SS were also demonstrated in patients with anterior dislocations [6, 45]. Marratt et al. (2015) [29] indicated that preoperative PT is strongly correlated with postoperative PT (*r*^*2*^ = 0.88; *P*-value = 0.0001). The preoperative sagittal anterior pelvic plane angle (APP) was the only predictive factor associated with a marked anterior or posterior change in PT [16]. Additionally, changes in SS, TK, and SVA parameters were correlated with changes in APP (*r*^*2*^ ≥ − 0.215; *P*-value ≤0.032) [16].

**Table 2:** Spinoplevic alignment parameters and LBP measured by the included papers

Study	Spinopelvic alignments and LBP																		
	CL	TK	LL	Scoliosis	LBP	SVA	SSA	T_**1**_spi	SS	PI	PT	PIA	TPA	APP	CA	Acetabular (lateral) inclination	Functional cup (acetabular) anteversion	Functional cup (acetabular) inclination	Pelvic motion
Lin et al., 2021		✓	✓			✓			✓	✓	✓				✓	✓			
Saiki et al., 2021			✓		✓	✓			✓	✓	✓								
Okuzu et al., 2021		✓	✓	✓	✓	✓			✓	✓	✓			✓					
Hagiwara et al., 2021			✓			✓			✓	✓									
Caglar et al., 2021		✓	✓	✓			✓		✓	✓	✓								
Ike et al., 2020									✓	✓						✓			
Homma et al., 2020			✓						✓	✓									
Klemt et al., 2020											✓						✓		
Can et al., 2020			✓		✓				✓	✓	✓								
Carender et al., 2020			✓						✓	✓	✓								
Cotter et al., 2020															✓	✓			
Kanto et al., 2019		✓	✓			✓			✓					✓					
Haws et al., 2019			✓						✓	✓	✓				✓				
Parilla et al., 2019			✓						✓	✓	✓								
Limmahakhun et al., 2019		✓							✓	✓	✓				✓		✓	✓	
Esposito et al., 2018			✓						✓										
Heckmann et al., 2018									✓						✓	✓			
Eguchi et al., 2018			✓	✓	✓				✓	✓	✓								
York et al., 2018									✓	✓	✓				✓	✓			
Piazzolla et al., 2018		✓	✓		✓	✓		✓	✓	✓	✓		✓						
Murphy et al., 2018											✓				✓	✓			
Okanoue et al., 2017																	✓		
Nam et al., 2017									✓		✓				✓	✓			
Ochi et al., 2017		✓	✓			✓			✓	✓	✓								
Eyvazov et al., 2016	✓	✓	✓		✓	✓			✓	✓	✓								
Abdel et al., 2016															✓	✓			
Furuichi et al., 2016			✓						✓					✓					
Tamura et al., 2016														✓					
Suzuki et al., 2016											✓								
Tripuraneni et al., 2016															✓	✓			
Weng et al., 2016			✓		✓			✓	✓	✓	✓								
Keshmiri et al., 2015																✓			
Maratt et al., 2015										✓	✓			✓	✓			✓	
Tezuka, 2014												✓			✓	✓			
Radcliff et al., 2013			✓						✓										
Murphy et al., 2013											✓								
Polkowski et al., 2012															✓				
Taki et al., 2012											✓								
Lazennec et al., 2011									✓						✓	✓			
Ishida et al., 2011											✓								
Zhu et al., 2010											✓								
Parvizi et al., 2010					✓														
Blondel et al., 2009										✓	✓								
DiGioia et al., 2006																			✓
Nishihara et al., 2003											✓								

*Abbreviations: APP* sagittal anterior pelvic plane, *CA* cup anteversion, *CI* cup inclination, *CL* cervical lordosis, *FI* functional inclination, *LBP* low back pain, *LL* lumbar lordosis, *PI* pelvic incidence, *PIA* pelvic inclination angle, *PT* pelvic tilt, *SS* sacral slope, *SSA* spinosacral angle, *SVA* sagittal vertical axis, *T*_*1*_*Spi* T_1_-spinopelvic inclination, *TK* thoracic kyphosis, *TPA* T_1_ pelvic angle

Decreased SS and lower PI are associated with increased risk of dislocation in patients with THA [56]. Patients with dislocations usually have lower SS and higher PI minus LL (PI-LL) compared to patients without dislocations [58]. Although Kanto et al. (2019) [16] reported that the SS was significantly decreased postoperatively (*P*-value = 0.003), Furuichi et al. (2016) [57] stipulated that impaired SS was improved in 41% of patients after THA.

The available literature suggests that body position can change spinopelvic alignments following THA. In a study conducted by Nam et al. (2017) [50], it has been also reported that the change in standing to sitting SS was significantly less in patients with a lumbar fusion (9.8° ± 8.2°) and history of prosthetic dislocation (12.5° ± 4.7°) versus patients without a history of lumbar surgery undergoing THA (*P*-value < 0.001 and *P*-value = 0.008). Tamura et al. (2016) [60] showed that PI in the sagittal plane was significantly lower in the standing position compared to the supine position (*P*-value < 0.01). Moreover, standing sagittal plane CA differed from supine anteversion by greater than 5° in more than 50% of patients in a study done by Polkowski et al. (2012) [48]. Tezuka (2014) [28] found similar results and mentioned that CA and CI were higher in the standing position compared to the supine position in patients after THA. It is worth mentioning that Tripuraneni et al. (2016) [61] did not find a significant difference in CI and CA between direct anterior and posterior approaches (*P*-value ≥0.12).

Functional cup anteversion (FCA) was another parameter measured by Okanoue et al. (2017) [31]. The angle was increased significantly over the 10-year follow-up compared to that at three weeks after surgery (*P*-value < 0.01). Preoperative posterior PT in the standing position and vertebral fractures after THA were significant predictors of increasing FCA (*P*-value ≤0.011) [31]. It has been also indicated there are marked differences in the relationship between FCA and PT in patients with severe lumbar degenerative disc disease compared with healthy control [51].

Patients with THA and LBP generally show increased TPA compared to patients without LBP (15.7° vs. -1.37°). LBP relief occurred in patients after THA [17]; however, Eyvazov et al. (2016) [32] declared that the improvement in LBP levels was not correlated with postoperative changes in spinopelvic alignment, including PI, PT, SS, CA, and CI (*P*-value ≥0.052). In a recent study, Okuzu et al. (2021) [39] concluded that among patients with LBP before THA, 62.9% had improved LBP at 1 year after THA. The preoperative factors associated with LBP improvement of LBP were a low Cobb angle (odds ratio [OR] = 0.95; 95% CI = 0.91–0.98); *P*-value < 0.01) and high APP angle (OR = 1.04; 95% CI = 1.00–1.08); *P*-value = 0.03) [39]. Moreover, patients with persistent LBP had a significantly lower APP angle (− 6.0° (10.3°)), lower LL (38.4° (20.7°)), greater SVA (45.2 mm (21.6–70.9 mm)), and greater PI-LL mismatch (9.3° (− 1.1° to 24.9°)) [39].

In a study performed in Japan, THA improved lumbar spine scoliosis since the Cobb angle was changed significantly from 45.81° to 43.70° in patients with unilateral hip OA [11]. Nevertheless, the results obtained from patients with bilateral hip OA showed that lumbar spine scoliosis did not change significantly after THA [preoperative angle = 5.50° (1.16°); postoperative angle = 3.73° (1.16°); *P*-value = 0.29)] [11].

Other sagittal spine alignments such as CL and TK were assessed in the available literature [32], and only TK had significant changes following THA (*P*-value = 0.042) [32]. Changes in spinopelvic alignments following THA with their relevant essential details are presented in Additional file 2.

### Methodological pitfalls among the included studies

Except for Klemt et al. (2020) [51] and Tripuraneni et al. (2016) [61], none of the selected studies employed a priori sample size calculations, and no post hoc power calculations were performed to determine whether the sample size was adequate to evaluate spinopelvic alignment changes and LBP following THA. The included studies did not specify the primary and secondary outcome measures, and the level of significance (α level) was not adjusted in the majority of outcome measures. Finally, the method of sampling was not indicated clearly in the majority of the studies, thereby the generalizability of results could be affected.

## Discussion

One of the main objectives of this scoping review was to assess the volume, yearly distribution, spinopelvic alignment parameters, and LBP measured in the published papers in the field of THA. Scoping reviews are a useful method when an overview is required to outline future research priorities by establishing what evidence is currently available [20, 68] or when limited evidence exists. This study highlights that a vast number of spinopelvic alignment parameters have been assessed in the published literature (Additional file 2). However, the results were not consistent across all studies for some spinopelvic alignment parameters.

We included in this scoping review 45 original studies published between 2003 and 2021. There has been a marked increase in publications since, with 73% of reviewed papers published between 2015 and 2021.

LBP was one of the outcomes measured in this study. Eguchi et al. (2018) [11] reported that severe hip OA may be associated with LBP and the authors demonstrated that THA could improve LBP in patients following THA. The authors suggested that the mechanism of LBP improvement following THA may be related to compensatory lumbar scoliosis improvement [11]. In addition, Eyvazov et al. (2016) [32] reported that changes in other spinopelvic alignment parameters were not correlated with LBP improvement in 28 patients after THA. However, the results of this study may be influenced by the limited sample size and high variability of the data [17]. Ben-Galim et al. (2007) [69], in a prospective cohort study on 25 patients undergoing THA, observed a significant improvement of LBP level without LL and sacral inclination changes. The authors of the current study noted that this lack of change may be related to the radiographic technique rather than to the actual clinical posture or gait [69].

Previous studies declared that changes in PT contribute to concomitant changes in the orientation of the acetabulum relative to the femur [29, 70, 71]. An increment in PT produces a functional increase in the anteversion of the acetabulum [58]. Conversely, a decrement in PT is accompanied by a functional decrease in acetabular anteversion [58]. For every degree added to PT, the typical acetabulum will gain 0.7° of anteversion [29, 70, 71]. This increase in acetabular anteversion, which inevitably follows an increase of PT between standing and sitting positions, helps clear the anterior lip of the acetabulum from impingement by the femoral neck, thus preventing posterior instability as the extreme range of flexion is approached [58].

Moreover, during sit-to-stand movement, especially in the extreme range of extension, there is a decrease in PT and subsequent functional retroversion of the acetabulum, clearing the posterior lip of the acetabulum from impingement by the femoral neck, thus preventing anterior instability [58]. However, a recent study did not observe a significant relationship between sagittal imbalance parameters and THA dislocations [30]. DelSole et al. (2017) [72], in a retrospective study, reported that patients who suffered a THA dislocation showed a greater PI-LL, but a normal CA was identified in 80% of patients. Although the authors reported a significant relationship between PT, SS, TPA, and T_9_ spinopelvic inclination with standing CA, the strength of associations was weak to moderate [72]. The results of the present study were interpreted based on the significance of *P*-values. A statistically significant correlation does not necessarily mean that the strength of the correlation is strong or high [73]. The *P*-value shows the probability that this strength may occur by chance [73]. The authors would have had to interpret the Pearson’s correlation coefficients based on their strength. Haws et al. (2019) [30], in a retrospective cohort study on 29 patients with THA dislocation, found similar results and revealed that spinopelvic sagittal parameters, including LL, PI, PT, SS, and PI-LL, were not associated with CA. They concluded that the relationship between spinal deformity and dislocation rates following THA may not be because of inaccurate cup orientation [30]. The lack of relationship between CA and spinopelvic sagittal balance in THA dislocations suggests that normal anteversion targets for acetabular cup placement may not be universally applicable [30, 62]. Therefore, controversy still exists regarding placement of the acetabular cup within the Lewinnek’s classic safe zone of 15° ± 10° of anteversion and postoperative dislocation risk [30, 62, 63].

York et al. (2018) [56], in a retrospective cohort study on 468 patients undergoing THA, showed that patients with lumbar spine fusion are at increased risk for postoperative dislocations. The authors of this study expressed that lower PI and SS in lumbar spine fusion patients compared to patients without prior lumbar spine fusion may predispose these patients to dislocation after THA [56]. Previous studies have shown that each additional level of spinal fusion decreased SS change from the standing to sitting position by 1.6° [56, 74]. More likely, the altered functional biomechanics of the lumbopelvic region increase the possibility of impingement or acetabular uncovering with subsequent dislocation [56, 75]. The sagittal orientation of the acetabular component is also related to total hip stability [76]. In a recent narrative review, Niemeier et al. (2020) pointed out that the increased CA places patients at increased risk of posterior impingement and anterior dislocation [76].

The sample size in the majority of the eligible studies was not defined a priori, and therefore future studies need to calculate sample size based on primary outcome(s) and probability of attrition rate depending on follow-up time. Moreover, the observed attrition rate should be adjusted during the statistical analysis through inverse probability weighting (IPW) [77] or multiple imputation (MI) [78]- two widely adopted approaches dealing with missing outcome data.

If a study contains outcome multiplicity, it is necessary to apply multiple comparison correction, which can greatly minimize the false positive errors [79]. Many standards of reporting (e.g.*,* CONSORT; Consolidated Standards of Reporting Trials) recommend that primary and secondary outcomes should be specified clearly with presentation of both estimated effect size and associated confidence interval to reduce the risk for selective outcome reporting.

Future studies could assess the relationship between spinopelvic alignment changes and LBP improvement following THA as the previously published studies on this topic had serious limitations. Besides, improvement in spinal column scoliosis after THA was not investigated in many studies, and scanty information exists about the mechanism of scoliosis improvement in patients undergoing THA. Previous studies mainly focused on the lumbar region of the spine; thus, there is a need for further research to explore alignment changes in upper regions of the spine (i.e.*,* cervical and thoracic) after THA.

### Limitations and strengths

Limitations of this scoping review must be acknowledged. First, we only assessed spinopelvic alignment and LBP following THA and studies in which the enrolled participants had undergone SRA were excluded from the review. Therefore, we need to bear in mind that these results cannot be generalized to patients with SRA. Second, our findings are dependent on information extracted from individual studies, all of which have their own methodological characteristics (e.g.*,* different study design and THA procedure) and objectives. Third, due to the nature of scoping reviews, we conducted a broad search with diverse search terms, which was a challenge. Finally, only four key databases were searched, and consequently, we may have missed some published articles. A key strength of this scoping review was its ability to provide a broad overview of spinopelvic alignment changes following THA. Also, in this scoping review, we highlighted the essential methodological limitations that exist in the studies of this area.

## Conclusions

This scoping review asked wide-ranging questions and investigated a diverse assortment of studies. Many studies reported that spinopelvic parameters such as PT and SS were changed following THA. However, the results were not consistent across the eligible studies. It has been demonstrated that patients with a THA dislocation had abnormal spinopelvic alignment compared to patients without THA dislocation. Furthermore, lumbar spine scoliosis and LBP were improved in patients undergoing THA. Several methodological issues were identified in the eligible studies, including no sample size calculation at the start of the study, no clear definition of primary and secondary outcomes, and no type-I error adjustment for multiple comparison conditions. Finally, some recommendations for future studies were provided in the last part of this paper.

## Supplementary Information

**Additional file 1.**

**Additional file 2.** Characteristics of the selected studies in chronological order.

## Data Availability

All data generated or analysed during this study are included in this published article [and its supplementary information files].

## Abbreviations

ADLs: activities of daily living
APP: anterior pelvic plane angle
CA: cup (acetabular) anteversion
CEBM: Oxford Centre for Evidence-Based Medicine
CENTRAL: Cochrane Central Register of Controlled Trials
CI: cup inclination
CONSORT: Consolidated Standards of Reporting Trials
FCA: functional cup anteversion
IPW: inverse probability weighting
IQR: interquartile range
LBP: low back pain
LL: lumbar lordotic
MeSH: medical subject heading
MI: multiple imputation
OA: osteoarthritis
PI: pelvic incidence
PI-LL: PI minus LL
PT: pelvic tilt
SRA: surface replacement arthroplasty
SS: sacral slope
SSA: Spinosacral angle
SVA: sagittal vertical axis
THA: total hip arthroplasty
TK: thoracic kyphosis
