# Supplementary material for: Spinopelvic alignment and low back pain after total hip arthroplasty: a scoping review
Source: BMC Musculoskelet Disord. 2022 Mar 15;23:250. doi: 10.1186/s12891-022-05154-7 (PMC8925238; doi:10.1186/s12891-022-05154-7)
Supplement: Supplementary file 1 — Additional file 1. [file 12891_2022_5154_MOESM1_ESM.docx]

| **Spinopelvic Alignment and Low Back Pain After Total Hip Arthroplasty**  A Scoping Review |
| --- |

**Additional Files**

**Table of Contents**

[Additional File 1: Search Strategies 3](#_Toc95507367)

[PubMed/MEDLINE (Last searched December 31, 2021) 3](#_Toc95507368)

[Scopus (Last searched December 31, 2021) 4](#_Toc95507369)

[Embase^®^ (Last searched December 31, 2021) 5](#_Toc95507370)

[Cochrane CENTRAL (Last searched December 31, 2021) 6](#_Toc95507371)

[Additional File 2: eTables 7](#_Toc95507372)

[eTable 1: Levels of evidence provided by different study types.^a^ 7](#_Toc95507373)

[eTable 2: List of excluded articles with reasons. 8](#_Toc95507374)

[eTable 3: Geographic locations where the studies were conducted and the journals in which the papers were published. 10](#_Toc95507375)

# **Additional File 1: Search Strategies**

## **PubMed/MEDLINE (Last searched December 31, 2021)**

(((“Hip replacement”[ti] AND arthroplasty*[ti]) OR (“hip prothesis”[ti] AND implantation*[ti]) OR (“hip”[ti] AND “Prosthesis implantation”[ti]) OR (“hip”[ti] AND “Prosthesis implantations”[ti]) OR (“Hip replacement”[ti] AND total[ti]) OR (“Hip replacements”[ti] AND total[ti]) OR (replacement*[ti] AND “Total hip”[ti]) OR (“total joint replacement”[ti] AND Hip[ti]) OR (“total joint replacements”[ti] AND Hip[ti]) OR “hip arthroplasty”[tiab] OR “hip joint alloplasty”[ti] OR “hip plasty”[ti] OR “hip extracapsular arthroplasty”[ti]) AND (Spin*[ti] OR deformit*[ti] OR Spinopelvic[ti] OR Spino-pelvic[ti] OR Alignment*[ti] OR Parameter*[ti] OR (Spinopelvic[ti] AND alignment*[ti]) OR (Spino-pelvic[ti] AND alignment*[ti]) OR (Spinopelvic[ti] AND parameter*[ti]) OR (Spino-pelvic[ti] AND parameter*[ti]) OR “pelvic tilt”[ti] OR “sacral slope”[ti] OR “pelvic incidence”[ti] OR Kyphosis[ti] OR Lordosis[ti] OR Scoliosis[ti] OR “sagittal vertical axis”[ti] OR “back pain”[ti] OR “Back pains”[ti] OR lumbago[ti] OR backache*[ti] OR “Range of Motion”[ti] OR (Joint[ti] AND flexibility[ti])))

## **Scopus (Last searched December 31, 2021)**

(((TITLE("Hip replacement") AND (arthroplasty*)) OR (TITLE("hip prothesis") AND (implantation*)) OR (TITLE("hip") AND ("Prosthesis implantation")) OR (TITLE("hip") AND ("Prosthesis implantations")) OR (TITLE("Hip replacement") AND (total)) OR (TITLE("Hip replacements") AND (total)) OR (TITLE(replacement*) AND ("Total hip")) OR (TITLE(“total joint replacement") AND (Hip)) OR (TITLE(“total joint replacements") AND (Hip)) OR TITLE-ABS(“hip arthroplasty”) OR TITLE(“hip joint alloplasty”) OR TITLE(“hip plasty”) OR TITLE(“hip extracapsular arthroplasty”)) AND (TITLE(Spin*) OR TITLE(deformit*) OR TITLE(Spinopelvic) OR TITLE(Spino-pelvic) OR TITLE(Alignment*) OR TITLE(Parameter*) OR (TITLE(Spinopelvic) AND (alignment*)) OR (TITLE(Spino-pelvic) AND (alignment*)) OR (TITLE(Spinopelvic) AND (parameter*)) OR (TITLE(Spino-pelvic) AND (parameter*)) OR TITLE(PT) OR TITLE("pelvic tilt”) OR TITLE("sacral slope") OR TITLE("pelvic incidence") OR TITLE(PI) OR TITLE(Kyphosis) OR TITLE(Lordosis) OR TITLE(Scoliosis) OR TITLE("sagittal vertical axis") OR TITLE("back pain") OR TITLE("Back pains") OR TITLE(lumbago) OR TITLE(backache*) OR TITLE("Range of Motion") OR (TITLE(Joint AND flexibility))))

- Document type was limited to article and conference papers
- Source type was limited to journals and conference proceedings

## **Embase^®^ (Last searched December 31, 2021)**

('hip replacement':ti AND arthroplasty*:ti OR ('hip prothesis':ti AND implantation*:ti) OR ('hip':ti AND 'prosthesis implantation':ti) OR ('hip':ti AND 'prosthesis implantations':ti) OR ('hip replacement':ti AND total:ti) OR ('hip replacements':ti AND total:ti) OR (replacement*:ti AND 'total hip':ti) OR ('total joint replacement':ti AND hip:ti) OR ('total joint replacements':ti AND hip:ti) OR ‘hip arthroplasty’:ti,ab OR 'hip joint alloplasty':ti OR 'hip plasty':ti OR 'hip extracapsular arthroplasty':ti) AND (spin*:ti OR deformit*:ti OR spinopelvic:ti OR 'spino pelvic':ti OR alignment*:ti OR parameter*:ti OR (spinopelvic:ti AND alignment*:ti) OR ('spino pelvic':ti AND alignment*:ti) OR (spinopelvic:ti AND parameter*:ti) OR ('spino pelvic':ti AND parameter*:ti) OR 'pelvic tilt':ti OR 'sacral slope':ti OR 'pelvic incidence':ti OR kyphosis:ti OR lordosis:ti OR scoliosis:ti OR 'sagittal vertical axis':ti OR 'back pain':ti OR 'back pains':ti OR lumbago:ti OR backache*:ti OR 'range of motion':ti OR (joint:ti AND flexibility:ti))

- Publication type was limited to article

## **Cochrane CENTRAL (Last searched December 31, 2021)**

(“hip replacement”:ti AND arthroplasty*:ti OR (“hip prothesis”:ti AND implantation*:ti) OR (“hip”:ti AND “prosthesis implantation”:ti) OR (“hip”:ti AND “prosthesis implantations”:ti) OR (“hip replacement”:ti AND total:ti) OR (“hip replacements”:ti AND total:ti) OR (replacement*:ti AND “total hip”:ti) OR (“total joint replacement”:ti AND hip:ti) OR (“total joint replacements”:ti AND hip:ti) OR “hip arthroplasty”:tiab OR “hip joint alloplasty”:ti OR “hip plasty”:ti OR “hip extracapsular arthroplasty”:ti) AND (spin*:ti OR deformit*:ti OR spinopelvic:ti OR “spino pelvic”:ti OR alignment*:ti OR parameter*:ti OR (spinopelvic:ti AND alignment*:ti) OR (“spino pelvic”:ti AND alignment*:ti) OR (spinopelvic:ti AND parameter*:ti) OR (“spino pelvic”:ti AND parameter*:ti) OR “pelvic tilt”:ti OR “sacral slope”:ti OR “pelvic incidence”:ti OR kyphosis:ti OR lordosis:ti OR scoliosis:ti OR “sagittal vertical axis”:ti OR “back pain”:ti OR “back pains”:ti OR lumbago:ti OR backache*:ti OR “range of motion”:ti OR (joint:ti AND flexibility:ti))

# Additional File 2: eTables

| **eTable 1:** Levels of evidence provided by different study types.^a^ | |
| --- | --- |
| **Level of evidence** | **Study type** |
| 1 | High-quality, well-designed and well-conducted RCT; systematic reviews or meta-analysis of these studies |
| 2 | Well-designed controlled trial without randomization; prospective comparative cohort/longitudinal study |
| 3 | Retrospective cohort/longitudinal study, case-control study, or systematic reviews of these studies |
| 4 | Case series with or without intervention; cross-sectional study |
| 5 | Expert opinion, case report, or bench research |
| **Abbreviation:** RCT: randomized clinical trial.  ^a^ Adapted from Oxford Centre for Evidence-Based Medicine (<http://www.cebm.net/2011-oxford-cebm-levels-evidence-introductory-document/>).  **Reference**  Ishii LE: **Thoughtful methods to increase evidence levels and analyze nonparametric data.** *JAMA Facial Plastic Surgery* 2015, 17:307-308. | |

| **eTable 2:** List of excluded articles with reasons. | | | |
| --- | --- | --- | --- |
| **#** | **Author(s)** | **Article title** | **Reason for exclusion** |
| 1 | An (2018) | Prior lumbar spinal fusion is associated with an increased risk of dislocation and revision in total hip arthroplasty: A meta-analysis | Meta-analysis paper |
| 2 | Barry and Hansen (2018) | The hip-spine relationship and its effect on total hip arthroplasty outcomes: What do the databases show? | Review paper |
| 3 | Brunner and Foucher (2018) | Sex specific associations between biomechanical recovery and clinical recovery after total hip arthroplasty | No measurement of spinopelvic alignment |
| 4 | Buckland (2017) | Dislocation of a primary total hip arthroplasty is more common in patients with a lumbar spinal fusion | No measurement of spinopelvic alignment |
| 5 | Buckland (2019) | Effects of sagittal spinal alignment on postural pelvic mobility in total hip arthroplasty candidates | Preoperative measurement of spinopelvic alignment |
| 6 | Buckland (2020) | Prevalence of sagittal spinal deformity among patients undergoing total hip arthroplasty | Preoperative measurement of spinopelvic alignment |
| 7 | De Leon (2020) | Spinopelvic instability in conversion total hip arthroplasty: A complicated case of loeys-dietz syndrome | Case report |
| 8 | DelSole (2017) | Total hip arthroplasty in the spinal deformity population: does degree of sagittal deformity affect rates of safe zone placement, instability, or revision? | Inclusion of patients with sagittal spinal deformity and concomitant THA |
| 9 | Diebo (2018) | Complications in patients undergoing spinal fusion after THA | No measurement of spinopelvic alignment |
| 10 | Eftekhary (2019) | A systematic approach to the hip-spine relationship and its applications to total hip arthroplasty | Review paper |
| 11 | Floman (1980) | Low-back pain and sciatica following total hip replacement: a report of two cases | Case report |
| 12 | Fritz (2021) | Is dislocation risk due to posterior pelvic tilt reduced with direct anterior approach total hip arthroplasty? | Different objectives |
| 13 | Haffer (2021) | Does obesity affect acetabular cup position, spinopelvic function and sagittal spinal alignment? A prospective investigation with standing and sitting assessment of primary hip arthroplasty patients | Different objectives |
| 14 | Heckmann and Lieberman (2021) | Spinopelvic biomechanics and total hip arthroplasty: A primer for clinical practice | Review paper |
| 15 | Ike (2018) | Spine-pelvis-hip relationship in the functioning of a total hip replacement | Review paper |
| 16 | Innmann (2019) | Can spinopelvic mobility be predicted in patients awaiting total hip arthroplasty? | No measurement of spinopelvic alignment |
| 17 | Innmann (2020) | Does spinopelvic mobility change following total hip arthroplasty? A prospective, diagnostic cohort study at one year following total hip arthroplasty | Conference proceeding |
| 18 | King (2018) | Time to dislocation analysis of lumbar spine fusion following total hip arthroplasty: Breaking up a happy home | No measurement of spinopelvic alignment |
| 19 | Kleeman-Forsthuber (2020) | Reliability of spinopelvic measurements that may influence the cup position in total hip arthroplasty | Reliability study |
| 20 | Langston (2018) | Risk factors for increased sagittal pelvic motion causing unfavourable orientation of the acetabular component in patients undergoing total hip arthroplasty | No measurement of spinopelvic alignment |
| 21 | Lazennec (2007) | Hip spine relationships: Application to total hip arthroplasty | No measurement of spinopelvic alignment |
| 22 | Luthringer and Vigdorchik (2019) | A preoperative workup of a “hip-spine” total hip arthroplasty patient: A simplified approach to a complex problem | No measurement of spinopelvic alignment |
| 23 | Madurawe (2021) | High prevalence of spinopelvic risk factors in patients with postoperative hip dislocations | Conference proceeding |
| 24 | Martz (2018) | Influence of body mass index on sagittal hip range of motion and gait speed recovery six months after total hip arthroplasty | No measurement of spinopelvic alignment |
| 25 | McKnight (2018) | Spinopelvic motion and impingement in total hip arthroplasty | No measurement of spinopelvic alignment |
| 26 | Morrison (1997) | Back pain, femoral vein thrombosis, and an iliopsoas cyst: unusual presentation of a loose total hip arthroplasty | Case report |
| 27 | Moskal (2020) | Dislocation rates following total hip arthroplasty via the direct anterior approach regardless of spinopelvic deformity | Conference proceeding |
| 28 | Nie (2017) | Gait kinematic deviations in patients with developmental dysplasia of the hip treated with total hip arthroplasty | No measurement of spinopelvic alignment |
| 29 | Niemeier (2020) | Sagittal pelvic kinematics in hip arthroplasty | Review paper |
| 30 | Nishiwaki (2018) | Pelvic tilt displacement before and after artificial hip joint replacement surgery | Different objectives |
| 31 | Perfetti (2017) | Prosthetic dislocation and revision after primary total hip arthroplasty in lumbar fusion patients: A propensity score matched-pair analysis | No measurement of spinopelvic alignment |
| 32 | Phan (2015) | The influence of sagittal spinal deformity on anteversion of the acetabular component in total hip arthroplasty | Review paper |
| 33 | Pierrepont (2017) | Variation in functional pelvic tilt in patients undergoing total hip arthroplasty | No measurement of spinopelvic alignment |
| 34 | Prakash (2002) | Does the lumbar spine need to be supported to stabilize the pelvis during total hip arthroplasty in the lateral position? | No data were presented |
| 35 | Renkawitz (2016) | Leg length and offset differences above 5 mm after total hip arthroplasty are associated with altered gait kinematics | No measurement of spinopelvic alignment |
| 36 | Rivière (2017) | The influence of spine-hip relations on total hip replacement: A systematic review | Review paper |
| 37 | Shah (2017) | Changes in spinopelvic indices after hip arthroplasty and its influence on acetabular component orientation | Not all patients had THA |
| 38 | Sharma (2021) | The hip-spine relationship in total hip arthroplasty: How to execute the plan | Annual Meeting Symposium |
| 39 | Shoji (2016) | Anterior inferior iliac spine bone morphology in hip dysplasia and its effect on hip range of motion in total hip arthroplasty | Inclusion of patients without THA |
| 40 | Stępiński (2021) | Spinopelvic alignment and its use in total hip replacement preoperative planning—decision making guide and literature review | Review paper |
| 41 | Tabata (2018) | Influence of hip center position, anterior inferior iliac spine morphology, and ball head diameter on range of motion in total hip arthroplasty | No measurement of spinopelvic alignment |
| 42 | Tanino (2018) | Hip stability after total hip arthroplasty predicted by intraoperative stability test and range of motion: A cross-sectional study | No measurement of spinopelvic alignment |
| 43 | Yang (2019) | The influence of pelvic tilt on the anteversion angle of the acetabular prosthesis | Review paper |
| 44 | Zeng (2015) | A randomized controlled trial: Preoperative home-based combined Tai Chi and Strength Training (TCST) to improve balance and aerobic capacity in patients with total hip arthroplasty (THA) | No measurement of spinopelvic alignment |
| 45 | Zhao (2021) | Does the change of acetabular anteversion result from lumbar pedicle subtraction osteotomy in ankylosing spondylitis-related kyphosis after primary total hip arthroplasty? | Different objectives |

| **eTable 3:** Geographic locations where the studies were conducted and the journals in which the papers were published. | |
| --- | --- |
| **Country** | **Study** |
| United States of America | [6, 35, 43, 46, 64, 66-68, 70-74, 76-78, 81-83, 86] |
| Japan | [3, 11, 16, 37, 47, 53, 55, 57, 59-61, 69, 75, 79, 80] |
| Turkey | [54, 84, 85] |
| France | [65, 87] |
| Germany | [56] |
| Italy | [17] |
| Canada | [58] |
| Taiwan | [62] |
| China | [88] |
|  |  |
| **Journal** | **Study** |
| Journal of Arthroplasty | [43, 57, 58, 61, 67, 72, 78-81] |
| Clinical Orthopaedics and Related Research | [66, 68-71, 77, 82, 83, 87] |
| European Spine Journal | [17, 54, 59, 88] |
| International Orthopaedics | [35, 60] |
| Orthopaedic Surgery | [16, 64] |
| Journal of Bone & Joint Surgery— *American Volume* | [6, 76] |
| The Bone & Joint Journal | [73] |
| Clinical Spine Surgery | [46] |
| Asian Spine Journal | [11] |
| HIP International | [47] |
| Kawasaki Medical Journal | [37] |
| Archives of Orthopaedic and Trauma Surgery | [56] |
| Japanese Journal of Joint Diseases | [53] |
| Journal of Orthopaedic Science | [55] |
| Orthopaedics & Traumatology: Surgery & Research | [65] |
| Acta Orthopaedica Et Traumatologica Turcica | [85] |
| Spine Deformity | [84] |
| Journal of Orthopaedic Research | [74] |
| Arthroplasty Today | [86] |
| Gait & Posture | [3] |
| BMC Musculoskeletal Disorders | [62] |
| Medicina | [75] |

| **eTable 4:** Relevant ongoing studies registered on ClinicalTrials.gov or WHO's International Clinical Trials Registry Platform. | | | | | |  |
| --- | --- | --- | --- | --- | --- | --- |
| **Register ID** | **Title** | **Register** | **Date of registration** | **Design** | **Outcome(s)** | **Recruitment status** |
| ChiCTR1900027859 | Postoperative dislocation after total hip arthroplasty in patients with Crowe type IV developmental dysplasia of the hip | ChiCTR | 30/11/2019 | Retrospective observational study | Diameter of acetabulum, CI | Recruiting (until 03/02/2020) |
| JPRN-jRCTs032180395 | Evaluation of usefulness of pelvic positioner with cup angle guide in total hip arthroplasty | JPRN | 20/03/2019 | Single arm interventional study | Accuracy of cup alignment | Recruiting (until 03/02/2020) |
| CTRI/2017/10/010084 | A single blind cluster randomized controlled trial to compare the clinical and radiological outcomes in patients with ankylosing spondylitis with fused hips and spinopelvic deformity undergoing total hip arthroplasty using a novel computer aided design (CAD) model based protocol vs. conventional technique | CTRI | 13/10/2017 | Cluster randomized trial | Pelvic parameters | Recruiting (until 03/02/2020 |
| NCT03261986 | A prospective RSA and clinical evaluation of the trident II acetabular cup | ClinicalTrials.gov | 23/08/2017 | Interventional study | Migration of the acetabular cup | Recruiting (until 03/02/2020) |
| ACTRN12617000063314 | Comparison of patient outcomes following total hip arthroplasty via an anterior or posterior approach: a prospective randomised controlled trial | ANZCTR | 12/01/2017 | Randomised parallel controlled trial | CA, CI | Recruiting (until 03/02/2020) |
| ACTRN12613000812796 | Dislocation, osteolysis, polyethylene wear, acetabular component migration and other complications 7 to 10 years following randomisation to either a large 36 mm or standard 28 mm diameter metal on highly cross-linked polyethylene articulation in total hip replacement | ANZCTR | 24/07/2013 | Interventional study | Acetabular component migration | Recruiting (until 03/02/2020) |
| ACTRN12609000958280 | A study to compare cluster hole and solid acetabular cups for lysis at five years following total hip arthroplasty in patients with osteoarthritis or rheumatoid arthritis | ANZCTR | 06/11/2009 | Interventional study | Cup migration | Recruiting (until 03/02/2020) |
| **Abbreviations:** CA: cup anteversion; CI: cup inclination. | | | | | | |
