# Supplementary material for: Spinopelvic alignment and low back pain after total hip arthroplasty: a scoping review
Source: BMC Musculoskelet Disord. 2022 Mar 15;23:250. doi: 10.1186/s12891-022-05154-7 (PMC8925238; doi:10.1186/s12891-022-05154-7)
Supplement: Supplementary file 2 — Additional file 2. Characteristics of the selected studies in chronological order. [file 12891_2022_5154_MOESM2_ESM.docx]

| **Additional file 2:** Characteristics of the selected studies in chronological order. | | | | | | | |  |
| --- | --- | --- | --- | --- | --- | --- | --- | --- |
| **#** | **Study** | **Aim(s)** | **Design** | **Population** | **Intervention** | **Spinopelvic outcome(s) measured** | **Key finding(s)** | **Level of evidence** |
| 1 | Lin et al., 2021 | 1. To analyze the interrelationship between global trunk alignment and spinopelvic motions in a group of patients who underwent THA 2. To identify spinopelvic motions with high risk to provide clinical guidance for component implantation | Retrospective study | 124 patients (48 males and 76 females); Average age of 65 years | THA;  anterior-lateral approach for 21 patients  (17%), posterior-lateral approach for  103 patients (83%). | TK, LL, SS, PT, PI, PI-LL, PFA, CA, CI, SVA  Position: Standing and upright relaxed sitting positions | In univariate regression analysis, ∆TK, ∆LL, and ∆PFA were correlated to ∆SVA. In multivariate regression analysis, ΔLL (*P* < 0.001) and ΔPFA  (*P* < 0.001) were found to be correlated to ΔSVA (ΔSVA=−11.97+ 0.05ΔTK – 0.23ΔLL – 0.17ΔPFA; adjusted *R^2^* =0.558). Spinopelvic stiffness was observed in 40 patients (32%), including five (4%) with paradoxical motion (∆PT=−3°(1)°, p < 0.001) with characteristics of balanced standing global trunk alignment (standing SVA=−1.0 (5.1)cm), similar stiffness of the lumbosacral spine (∆LL=−7°±5°), higher hip motion (∆PFA=−78°±6°, *P* =0.017), and higher anterior trunk shift (∆SVA=6.2 (2.0)cm, *P* =0.003) from standing to sitting as compared to the stiffness group. | 3 |
| 2 | Saiki et al., 2021 | To evaluate how changes in spinal malalignment correlate with improvement in preoperative LBP in patients who underwent THA for hip OA | Prospective  study | 77 patients (14 male, 63 female) who underwent primary THA; Average age 62 (range 28-83) years | THA; posterior approach | C_7_-SVA, LL, PI, PT, SS, PI-LL, pelvic obliquity, LBP, C_7_-central sacral vertical line  Position: Standing  Radiographic data were obtained before surgery and 1 year after surgery | The sagittal parameters changed slightly but significantly (except C7-SVA and PI-LL). Coronal parameters, including pelvic obliquity and C_7_-central sacral vertical line significantly improved. Twenty-six (37%) patients had LBP preoperatively. Patients with LBP had smaller LL, larger PT, and larger PI-LL than the patients without LBP. Fourteen (54%) of the 26 patients with preoperative LBP showed pain improvement, but there were no significant differences in the radiographic parameters. | 2 |
| 3 | Okuzu et al., 2021 | 1. To determine the proportion of patients with improved LBP after THA 2. To identify the preoperative spinal factors associated with LBP improvement | Retrospective study | 151 patients who underwent primary THA and had a preoperative VAS score for LBP of ≥ 2; Average age 62.9 (13.5) years | THA; antero-lateral approach with or without mini trochanteric osteotomy of the anterior portion of the gluteus medius insertion | PT, SS, LL, PI, TK, SVA, PI-LL, APP, Cobb angle, LBP, pelvic obliquity*  * Pelvic obliquity was defined as the angle between the line connecting the bilateral top of the ilium and the horizontal line  Position: Spine radiographs were examined while patients stood in a relaxed position with a horizontal gaze | Among the coronal spinal parameters, the Cobb angle was significantly lower in the LBP improved group. Among the sagittal spinal parameters, the LBP continued group showed a significantly more posteriorly titled pelvis, lower LL, greater SVA, and greater PI-LL mismatch, indicating a sagittal spinal imbalance. Logistic regression analysis found that preoperative factors associated with LBP improvement after THA had a low Cobb angle (OR = 0.95; 95% CI = 0.91-0.98); *P*-value <0.01) and high APP angle (anteriorly tilted pelvis) (OR = 1.04; 95% CI = 1.00-1.08); *P*-value = 0.03). | 3 |
| 4 | Hagiwara et al., 2021 | To examine the risk factors of impingement that can lead to dislocation using pre- and postoperative spinopelvic radiographs | Prospective  study | 95 patients (22 male, 73 female) with end-stage hip osteoarthritis who were eligible for THA; Average age 62.2 (12.8) years | THA; direct anterior approach with traction table in a  Supine position. | PI, SS, LL, SVA  Position: Standing and sitting lateral radiographs  Radiographic data were obtained within 1 month prior to and 6 months after THA | **SS**  Preoperative: standing 30.3°±9.9°; sitting 16.4°±10.3°, *P* = <0.001  Postoperative: standing 28.0°±9.9°*; sitting 16.0°±11.0°, *P* = <0.001  * Statistically significant between Pre-op and Post-op (*P* = 0.01)  **LL**  Preoperative: standing 37.8°±12.8°; sitting 21.7°±14.0°, *P* = <0.001  Postoperative: standing 36.7°±13.0°; sitting 21.7°±12.9°, *P* = <0.001  **SVA**  Preoperative: standing 38.4mm±38.9mm; sitting 60.6mm± 31.1mm, *P* = <0.001  Postoperative: standing 35.3mm±37.6mm; sitting 58.3mm± 27.3mm, *P* = <0.001  A multiple linear regression analysis showed that the PI–LL, and LL changes were associated with the posterior impingement (*β* = − 0.21, and 0.24, respectively). Moreover, PI–LL was also associated with the anterior impingement in the sitting position (*β* = − 0.27). The results suggest that a flatback with a rigid spine leads to posterior impingement, and a well-balanced spine leads to anterior impingement, which can be a potential risk factor for dislocation. | 2 |
| 5 | Caglar et al., 2021 | To investigate the effect of THA with femoral shortening on spinopelvic parameters in patients with  neglected high hip dysplasia | Retrospective study | 20 female patients diagnosed with Crowe 3 or 4 NHD were identified to have undergone THA with femoral shortening; Average age 51.6 (range 31–64) years | THA with femoral shortening | SS, PI, PT, SSA, Cobb angle, segmental LL, segmental TK  Position: Full spine standing  anteroposterior and lateral radiographs  Radiographic data were obtained before surgery and at a minimum follow-up time of one year (mean 17.4 months) | **Preoperative mean values of spinopelvic parameters**  T12–L1: 4.3°±3.5°; L1–L2: 7.8°±4.6°; L2–L3: 13.6°±6.2°; L3–L4: 17.2°±5.0°; L4–L5: 23.6°±5.6°; L5–S1: 26.5°±6.4°; T2–T5: 11.9°±6.0°; T5–T12: 26.0°±8.5°; T10–T12: 5.5°±4.0°; T10–L2: 7.3°±6.3°; L4–S1: 43.1°±7.2°; T1–T12: 39.4°±8.5°; L1–S1: 68.7°±9.7°; SS: 52.1°±8.9°; Cobb angle: 12.3°±8.4°; SSA: 146.4°±6.6°.  **Postoperative mean values of spinopelvic parameters**  T12–L1: 4.0°±2.2°, *P* = 0.742; L1–L2: 8.7°±5.1°, *P* = 0.253; L2–L3: 14.5°±7.2°, *P* = 0.498; L3–L4: 17.2°±5.7°, *P* = 0.913; L4–L5: 25.6°±7.4°, *P* = 0.488; L5–S1: 24.8°±6.7°, *P* = 0.177; T2–T5: 14.3°±6.6°, *P* = 0.15; T5–T12: 26.8°±9.6°, *P* = 0.431; T10–T12: 3.8°±2.8°, *P* = 0.175; T10–L2 : 9.7°±5.9°, *P* = 0.14; L4–S1: 40.6°±6.9°, *P* = 0.7; T1–T12: 40.4°±10.8°, *P* = 0.519; L1–S1: 66.9°±8.7°, *P* = 0.432; SS: 52.5°±7.3°, *P* = 0.911; PT: 6.5°±9.3°; PI: 57.9°±9.7°; Cobb angle: 9.2°±5.5°, *P* = 0.02. | 3 |
| 6 | Ike et al., 2020 | To investigate the influence of PI on the sagittal position of the hip joint and whether the PI created a risk of impingement | Prospective study | 187 patients (200 hips) who underwent primary THA; Average age 51.6 (range 31–64) years | THA; with computer navigation used for placement of the cup | PI, SS, PFA, AI, CSI (AI + PFA) | The PI was normal in 145 hips (73%), low in 18 hips (9%), and high in 37 hips (19%). Eighty-two hips had spinopelvic imbalance: 12 (67%) of the 18 hips with low PI, 56 (39%) of the 145 hips with normal PI, and 14 (38%) of the 37 hips with high PI. Low-PI hips was the most predictive of the risk of impingement and postoperatively these hips had the most outliers from the functional safe zone. | 2 |
| 7 | Homma et al., 2020 | 1. To elucidate the statistical characteristics of preoperative and post-operative pelvic mobility 2. To investigate the relationship between pre-operative spinopelvic factors and postoperative pelvic mobility | Retrospective study | 86 patients (15 male, 71 female) with HOA who underwent primary THA; Average age 67.4 (10.1) years | THA; direct anterior approach in the supine position | Pelvic mobility (SS_standing_ - SS_sitting_), LL, PI  Position: Sagittal spinopelvic alignment was assessed on standing and sitting lateral radiographs of the full spine  Radiographic data were obtained before THA (preoperative period) and three months after THA (postoperative period) | The median (IQR) preoperative and postoperative pelvic mobility was 19.0° (13.75°–27.0°) and 16.0° (10.0°–25.25°), respectively, with significant difference. The pre-operative SS while standing and preoperative pelvic mobility were associated with postoperative pelvic mobility (*r* = 0.409, *P* < 0.05 and *r* = 0.533, *P* < 0.05). The multivariate linear regression analysis showed that the following factors contributed to post-operative pelvic mobility: incidence of osteoarthritis in the contralateral hip, lumbar scoliosis, preoperative SS while standing, and preoperative SS while sitting. | 3 |
| 8 | Klemt et al., 2020 | To investigate the in vivo association of lumbar DDD with the PT angle and with FCA during postural changes in THA patients | Observational study | 50 patients with unilateral THA; Average age 62.5 (8) years | THA; posterior lateral approach | PT, FCA, ΔPT, ΔFCA  Position: Data were measured during supine, standing, swing-phase, and stance-phase positions using a validated dual fluoroscopic imaging system | The authors concluded that PT, FCA, ΔPT, and ΔFCA were significantly correlated with the severity of lumbar DDD. Patients with severe lumbar DDD showed marked differences in PT with changes in posture; there was an anterior tilt (-16.6° vs -12.3°, *P* = 0.047) in the supine position, but a posterior tilt in an upright posture (1.0° vs -3.6°, *P* = 0.005). A significant decrease in ΔFCA during stand-to-swing (8.6° vs 12.8°, *P* = 0.038) and stand-to-stance (7.3° vs 10.6°,*P* = 0.042) was observed in the severe lumbar DDD group. | 2 |
| 9 | Can et al., 2020 | 1. Changes in the sagittal alignment after THA in patients with HOA secondary to Crowe type-IV DDH 2. Whether THA would contribute to the relief of LBP | Retrospective study | 27 patients (2 male, 25 female) with bilateral HOA secondary  to Crowe type-IV DDH; Average age 40.36 (12.35) years | THA; simultaneous, bilateral THA | PI, SS, LL, PT, LBP  Position: Standing anteroposterior and  lateral lumbosacral radiographs and pelvis anteroposterior radiographs  Radiographic data were obtained before surgery and at a minimum follow-up time of 24 months | PT and PI significantly changed from -20.4°±20.4° to 3.2°±16.7° (*P* = 0.001) and from 26.6°±35.1° to 47.4°±17.9° (*P* = 0.001), respectively. There were no significant differences regarding the SS and LL measurements (SS, 46.9°±11.9° to 44.1°±11.3°, *P* = 0.335; LL, 65.9°±12.9° to 61.2°±14.1°, *P* = 0.084).  Preoperatively, all patients had LBP. Preoperative and final follow-up ODI scores were 48.3 and 3.9 (*P* = 0.000), respectively. | 3 |
| 10 | Carender et al., 2020 | To identify the prevalence  of abnormal spinopelvic relationships in patients presenting for primary THA | Retrospective study | 228 patients (127 male, 101 female) with THA; Average age 60.0 (13.1) years | THA; | LL, PI, SS, PT, PI-LL  Position: Sitting and standing anteroposterior and  lateral radiographs | The prevalence of spinopelvic imbalance was 62.3%, the prevalence of decreased spinopelvic motion was 34.2%, and the prevalence of both spinopelvic imbalance and decreased spinopelvic motion was 22%.  Mean values of radiographic measurements on standing and sitting radiographs:  **Standing**  LL: 43.5° ± 14.7°; PI: 57.6° ± 12.1°; SS: 42.1° ± 11.8°; PT: 15.3° ± 8.8°; PI-LL: 14.1° ± 13.7°  **Sitting**  SS: 26.5° ± 12.2°  **ΔStanding to sitting**  SS: 15.6° ± 13.1° | 3 |
| 11 | Cotter et al., 2020 | To determine if there is a significant change in CI and CA over a 10-year period following THA | Retrospective study | 46 patients (17 male, 29 female) with THA; Median age 56 (48-62) years | THA; most cases were performed with the lateral approach, 3 had a posterior approach and 1 had a direct  anterior approach | CI, CA, sacro-femoral-pubic angle*  Position: Supine anteroposterior pelvic  Radiographs  Radiographic data were obtained after surgery and at a minimum follow-up time of 10 years  * It is defined as the angle between a line from the center of the femoral head  to the midpoint of S1 endplate and to the pubic symphysis on the AP pelvic radiograph. | The study found no significant change in functional cup orientation a minimum of 10 years after THA. No shifts in functional cup orientation as a result of altering spinopelvic alignment seemed to be present over a 10-year period.  **CI**  Initial postoperative: median 47° (IQR 43°-51°)  Minimum of 10 y following surgery: median 46° (42°-50°)  Change: -1° (-3° to 2°)  **CA**  Initial postoperative: median 20° (IQR 10°-25°)  Minimum of 10 y following surgery: median 20° (11°-25°)  Change: 0 (-1 to 3) | 3 |
| 12 | Kanto et al., 2019 | 1. Anterior or posterior changes in PT was present after THA 2. Correlation between anterior or posterior change in PT and spinopelvic parameters after THA | Prospective study | 100 patients (15 male, 85 female) with THA; Average age 61 (15) years | THA; modified Hardinge  approach in a lateral position | LL, TK, APP, SVA, SS  Position: Relaxed upright standing posture with the hips and knees as fully extended as possible  Radiographic data were obtained preoperatively and 1 year after THA | Changes in PT was observed in 59 patients (59%) postoperatively, while PA was observed in 16 patients (16%) and PR was observed in 25 patients (25%). There were no significant differences between preoperative and postoperative LL (46.6⁰ (14.3⁰) *vs* 45.2⁰ (14.9⁰), *P* = 0.289), TK (25.9⁰ (10.9⁰) *vs* 25.7⁰ (11.9⁰), *P* = 0.836), and SVA (21.0⁰ (36.6⁰) *vs* 23.2⁰ (36.0⁰), *P* = 0.219). However, the SS (38.4⁰ (9.7⁰) *vs* 37⁰ (9.9⁰), *P* = 0.003) was significantly decreased postoperatively.  **Correlation between Δ APP and change in spinopelvic Parameters:**  Δ LL, *r* = −0.015, *P* = 0.88; Δ TK, *r* = −0.215, *P* = 0.032; Δ SS, *r* = 0.237, *P* = 0.031; Δ SVA, *r* = 0.242, *P* = 0.015. | 2 |
| 13 | Haws et al., 2019 | To assess the effect of spinal sagittal alignment on cup anteversion in THA dislocation | Retrospective study | 29 patients with THA dislocation. 18 (62%) patients had undergone a primary THA, whereas 11 (38%) had undergone a revision THA | THA; lateral (19 patients; 65.52%), anterior (5 patients; 17.24%), and posterior (5 patients; 17.24%) approaches | LL, PI, PT, SS, PI-LL difference, CA, correlation of CA with sagittal parameters  Position: Cross-table lateral hip radiographs and lateral upright lumbar spine radiographs | A majority of patients with a THA dislocation showed abnormal sagittal balance:  LL: 47.68⁰ (17.22⁰); PI: 61.53⁰ (12.89⁰); PT: 22.28⁰ (11.41⁰); SS: 39.70⁰ (9.88⁰), PI-LL difference: 17.65⁰ (15.44⁰);  normal PI-LL difference (< 10⁰): 9 patients (31.03%); abnormal PI-LL difference (≥ 10⁰): 20 patients (68.97%); CA: 25.46⁰ (14.71⁰); normal CA (15⁰ (10⁰)): 11 patients (37.93%); abnormal cup anteversion: 18 patients (62.07%).  However, sagittal balance was not correlated with CA:  LL, *r* = 0.085, *P* = 0.66; PI, *r* = 0.175, *P* = 0.365; PT, *r* = 0.252, *P* = 0.188; SS, *r* = −0.099, *P* = 0.611; PI-LL difference, *r* = −0.011, *P* = 0.956. | 3 |
| 14 | Parilla et al., 2019 | Relationships between spinopelvic measurements and post-THA hip instability and to determine if procedure order reveals a difference in hip dislocation rate | Retrospective study | 135 patients met inclusion criteria, 73 underwent  LSF followed by THA and 62 underwent THA followed by LSF. However, 89 patients had complete radiograph series | THA, and LSF | LL, PT, PI, SS, PI-LL difference  Position: Not clearly described  Radiographic data were accessed preoperatively, immediately postoperatively, 3 and 6 months, and 1 year postoperatively, and, when possible, 1.5 and 2 years postoperatively | No significant differences in dislocation rates between operative order groups were elicited (7/73 LSF first, 4/62 THA first; *P* = 0.509). Compared to non-dislocators, dislocators had lower LL (−10.9°) and SS (−7.8°), and higher PT (+4.3°) and PI-LL (+7.3°). Additional risk factors for dislocation included sacral fusion (relative risk [RR] = 3.0) and revision fusion (RR = 2.7). | 3 |
| 15 | Limmahakhun et al., 2019 | To compare PT, anteversion, inclination, and position of head-cup contact points in patients with lumbar DDD and a matched patient cohort without DDD | Observational analytical case-controlled study | 50 hips (43 patients) with THA | THA; posterior lateral  approach | FCA, PI, PT, SS, LL, CA, FCI  Data were measured during supine, standing positions using a validated dual fluoroscopic imaging system | Patients with lumbar DDD demonstrated decreased maximum (5.9˚ ± 4.2˚ vs. 9.3˚ ± 5.4˚, *P* = 0.02) and minimum (2.4˚ ± 4.1˚ vs. 6.2˚ ± 5.6˚, *P* = 0.01) anterior PT, and increased maximum CA (29.3˚ ± 8.7˚ vs. 25.1˚ ± 8.1˚, *P* = 0.05). | 2 |
| 16 | Esposito et al., 2018 | To determine whether patients who dislocate following THA have different sitting spinopelvic alignment or acetabular component orientation compared to patients who did not dislocate | Cross-sectional study*  * It seems that the present study was a cohort study rather than a cross-sectional study | 158 patients with THA | THA; posterior approach | Standing LL, sitting LL, standing SS, sitting SS, spine flexion, change in SS from standing to sitting  Position: Standing and  sitting radiographs from the thoracolumbar junction (T12-L1) to  the ankles  Radiographic data were obtained preoperatively and 1 year after THA | Patients who dislocated had significantly less spine flexion and less change in PT from standing to sitting positions compared to patients with normal spines. In sitting position, dislocators had acetabular components with less functional inclination and less functional anteversion.  **Normal Spine Non-dislocator (*n* = 106):**  Standing LL: 58° (10°); sitting LL: 35° (16°); standing SS: 40° (8°); sitting SS: 23° (11°); spine flexion: 23° (13°); change in SS from standing to sitting: 17° (10°);  **DDD Spine Non-dislocator (*n* = 40):**  Standing LL: 47° (13°); sitting LL: 34° (14°); standing SS: 32° (10°); sitting SS: 23° (10°); spine flexion: 12° (9°); change in SS from standing to sitting: 9° (8°);  **Normal Spine Dislocator (*n* = 1):**  Standing LL: 57°; sitting LL: 25°; standing SS: 33°; sitting SS: 6°; spine flexion: 32°; change in SS from standing to sitting: 27°;  **DDD Spine Dislocator (*n* = 11);**  Standing LL: 49° (11°); sitting LL: 35° (10°); standing SS: 34° (6°); sitting SS: 25° (8°); spine flexion: 14° (8°); change in SS from standing to sitting: 9° (6°);  **All** T**HA patients (*n* = 158):**  Standing LL: 54° (12°); sitting LL: 35° (10°); standing SS: 38° (9°); sitting SS: 23° (10°); spine flexion: 20° (13°); change in SS from standing to sitting: 15° (10°). | 2 |
| 17 | Heckmann et al., 2018 | To assess the mechanical causes of late dislocation, including the influence of spinopelvic motion | Cohort study | 20 patients with THA | THA; posterior approach | Standing SS, sitting SS, change in SS from standing to sitting, standing cup ante-inclination, sitting cup ante-inclination  Position: Lateral standing and sitting spine-pelvis-hip radiographs were used  Radiographic data were obtained after 1 year following primary THA | Patients with an anterior dislocation had spinopelvic abnormalities such as fixed posterior PT when standing. The mean CA in the anterior group was 19.1⁰ (1.9⁰), and the mean inclination was 45.8⁰ (2.3⁰). However, patients with a posterior dislocation had abnormal spinopelvic measurements such as decreased spinopelvic motion defined by a Δ SS of ≤10⁰ (range, 22⁰ to 10⁰). | 2 |
| 18 | Eguchi et al., 2018 | To examine change in lumbrosacral spine alignment and LBP following THA in patients with severe hip osteoarthritis | Retrospective observational study | 30 patients (1 male, 29 female) with THA; Average age 63.5 years [range 45–78 years] | THA; anterior approach | Lumbar scoliosis, SS, LL, PT, PI, LBP  Position: Lumbosacral spinal alignment measurements from standing radiographs were performed  Radiographic data were obtained preoperatively, 1 month postoperatively, and periodically until the final observation (average 126.9 days) | **Patients with unilateral THA:**  LBP (using VAS): mean change = -16.42 mm; *P* = 0.016, LS: mean change = -3.09°; *P* = 0.037, SS: mean change = -2.01°; *P* = 0.46, LL; mean change = -2.46°; *P* = 0.61, PT: mean change = -0.39°; *P* = 0.84, PI: mean change = -1.39°; *P* = 0.58.  **Patients with bilateral THA:**  LBP (using VAS): mean change = -17.17 mm; *P* = 0.07, LS: mean change = -1.77°; *P* = 0.29, SS: mean change = -2.84°; *P* = 0.59, LL; mean change = -1.58°; *P* = 0.83, PT: mean change = 1.48°; *P* = 0.66, PI: mean change = -3.69°; *P* = 0.52. | 3 |
| 19 | York et al., 2018 | 1. To determine if LSF increases the risk of dislocation following THA 2. To investigate anatomic variables associated with this increased risk. | Retrospective study | 509 primary THAs in 460 patients (60% male); Age range 35–95 years | THA; posterior approach | PI, PT, SS, anteversion, lateral inclination  Position: Acetabular component lateral inclination and anteversion positions were measured from standing antero-posterior radiographs. SS, PT, and PI were measured from lateral imaging | **Dislocators (*n =* 9) *vs.* non-dislocators (*n* = ):**  PI: 45.2 *vs.* 58.6 (***n* = 17**); *P* = 0.0029  PT: 19.1 *vs.* 23 (***n* = 17**); *P* = 0.436  SS: 26.2 *vs.* 36.5 (***n* = 17**); *P* = 0.0384  Anteversion: 23.99 *vs.* 27.95 (***n* = 22**); *P* = 0.397  Lateral inclination: 38.3 *vs.* 40.5 (***n* = 22**); *P* = 0.4696  Lower PI and decreased SS are associated with increased risk of dislocation in patients with LSF. | 3 |
| 20 | Piazzolla et al., 2018 | To assess the relationship between femoral neck  anteversion, LBP, and spinopelvic parameters in patients undergoing THA for unilateral severe primary HOA | Prospective study | 91 patients  (57 females, 34 males) with THA; Average age 65.8 years [ range 56–78 years] | THA; lateral Hardinge approach | PI, PT, SS, LL, TK, T_1_Spi, TPA, SVA, LBP  Position: Full spinal X-rays in upstanding position were performed  Radiographic data were obtained preoperatively and after 6 months following primary THA | **Patients with THA and LBP:**  PT: 8.8° (8.48°); PI: 49.27° (8.84°); SS: 41.13° (5.59°); LL: -52.93° (6.74°); TK: 55.07° (9.3°); T1Spi: -2.2° (3.7°); TPA: 15.7° (8.9°); SVA: 5.973° (1.57°)  **Patients with THA without LBP:**  PT: 13.3° (7.5°); PI: 46.3° (9.4°); SS: 38.3° (6.2°); LL: 45.63° (4.84°); TK: 52.07° (7.46°); T1Spi: -1.34° (4.3°); TPA: -1.37° (4.45°); SVA: 3.5° (3.23°)  After THR, a relief of both hip and LBP and a change in spinopelvic parameters was observed. | 3 |
| 21 | Murphy et al., 2018 | To measure CA and CI in stable and unstable hips to  assess the presence of a safe zone for cup position in THA | Retrospective study | 34 patients who had undergone revision of the acetabulum for recurrent instability and 175 patients with stable THA; Average age 64.5 (11) years [range 20–90 years] | THA | CA, CI, PT  Position: Coronal and sagittal CT images were taken | Anteriorly unstable hips compared with stable hips had significantly higher operative anteversion of the cup (44° ± 12° versus 31° 6 11°, respectively; mean difference, 13°; 95%CI, 5°-21°; *p* = 0.003). Posteriorly unstable hips compared with stable hips had lower operative anteversion of the cup (19° ± 15° versus 31° ± 11°, respectively; mean difference, -12°; 95% CI, -5° to -18°; p = 0.001) and pelvic tilt (0° ± 6° versus 4° ± 6°, respectively; mean difference, -4°; 95% CI, -7° to -1°; *p* = 0.007).  In addition, a safe zone with no unstable hips was identified within 42° 6 12° of operative CI and 31° 6 8° of operative CA. | 3 |
| 22 | Okanoue et al., 2017 | To clarify the chronological changes in functional cup position at a minimum follow-up of 10 years after THA | Retrospective study | 58 patients (6 male, 52 female) with THA; Average age 67 years (range 36-81 years) | THA; direct lateral approach | FCA  Position: Anteroposterior pelvic radiographs in the supine and standing positions. Also, lateral pelvic radiographs in the supine and standing positions were obtained  Radiographic data were obtained before, 3 weeks after, and every 1 year after surgery | FCA angle was increased significantly over the 10-year follow-up compared to that at 3 weeks after surgery (*P* < 0.01). The maximum postoperative change in FCA was 17.0° anteriorly; 12 patients with THA (21%) showed a postoperative change in FCA by >10° anteriorly. Moreover, preoperative posterior PT in the standing position and vertebral fractures after THA were significant predictors of increasing FCA. | 3 |
| 23 | Nam et al., 2017 | To determine the impact of THA implantation on pelvic motion, and to assess motion in  patients with a history of lumbar fusion or prosthetic dislocation | Prospective study | 1) patients without a history of lumbar surgery undergoing THA (Group A, *n* = 24), 2) patients with a lumbar fusion (Group B, *n* = 27), and 3) patients with a THA prosthetic dislocation (Group C, *n* = 7) | THA; mini-posterior approach | SS, PT, CA, CI  Position: All patients received both standing and sitting lateral pelvis images to measure sacral slope and pelvic tilt in the sagittal plane | **Standing SS:** Group A: 42.3° ± 11.4°; Group B: 36.5° ± 9.5°; Group C: 36.5° ± 9.5°  **Sitting SS:** Group A: 20.3° ± 11.6°; Group B: 26.7° ± 12.9°; Group C: 23.7° ± 13.2°  **Standing Sagittal PT:** Group A: 13° ± 8.1°; Group B: 19.3° ± 8.5°; Group C: 23° ± 13.7°  **Sitting Sagittal PT:** Group A: 37.7° ± 13.5°; Group B: 30.9° ± 9.2°; Group C: 33° ± 13.2°  **Difference between Standing and Sitting SS:** Group A: 22.1° ± 15.2°; Group B: 9.8° ± 8.2°; Group C: 12.5° ± 4.7°  **CI:** Group B: 37.7° ± 6.3°; Group C: 40.4° ± 6.5°; *P* = 0.8  **CA**: Group B: 34.9° ± 18.2°; Group C: 35.6° ± 10.6°; *P* = 0.7 | 2 |
| 24 | Ochi et al., 2017 | To investigate the association between preoperative sagittal spinopelvic alignment and postoperative clinical outcomes after THA | Retrospective study | 197 patients (15 male, 77 female) who had undergone THA; Average age 67.5 (10.1) years | THA; direct anterior approach | SVA, TK, LL, PT, PI, SS, PI-LL  Position: Sagittal spinopelvic alignment was assessed on standing lateral radiographs of the full spine, including the pelvis and femoral heads  Radiographic data were obtained before and 3 to 24 months after surgery | Patients with preoperative imbalanced sagittal alignment such as larger SVA, larger PI-LL and retroversion of pelvis had poorer clinical outcomes than others after THA. While those preoperative imbalanced patients with anteversion of pelvis may have a compensatory ability which could correct the abnormal sagittal alignment after THA. Preoperative sagittal spinopelvic alignment affected postoperative clinical outcomes after THA | 3 |
| 25 | Eyvazov et al., 2016 | To investigate postoperative changes in spinal sagittal alignment in patients with hip spine syndrome and to verify whether any correlation exists between these changes and improvement of LBP symptoms following THA | Prospective study | 28 patients (11 male and 17 female) with hip-spine syndrome who underwent  THA surgery; Average age 61.7 (6.4) years (range 52–76 years) for males and 60.3 (7.9) years (range 50–75 years) for females | THA; anterolateral approach | CL, TK, LL, PI, PT, SS, SVA, LBP  Position: Long-cassette anteroposterior and lateral standing X-rays were obtained  Radiographic data were obtained before surgery and at 6 months after surgery | **Preoperative; Median (IQR):**  PI: 50° (35°, 60°); PT: 11° (7°, 23°); SS: 36° (31°, 42°); CL: 14° (7°, 24°);  TK: 45° (38°, 55°); LL: 49° (41°, 68°); SVA: 5mm (-11mm, 41mm)  **Postoperative; Median (IQR):**  PI: −; PT: 12° (8°, 17°); SS: 37° (29°, 41°); CL: 13° (11°, 26°);  TK: 44° (31°, 55°); LL: 55° (44°, 60°); SVA: 0mm (-20mm, 28mm)  No significant changes have been noted in radiographic spinal sagittal alignment postoperatively except for TK (*P* = 0.042). Furthermore, the improvement in LBP levels was not correlated with postoperative changes in spinopelvic alignment. | 2 |
| 26 | Abdel et al., 2016 | To determine if the  radiographic target values for cup inclination and anteversion proposed as the Lewinnek et al. safe zone accurately  predicted dislocation in a contemporary THA practice | Retrospective study | 206 patients THA (143 male and 63 female) with dislocated; Average age 63 (16.1) years [range 17–94 years] | THA; anterolateral, posterior, extended trochanteric  trochanteric osteotomy | CI, CA  Position: Radiographic analysis included inclination and anteversion of the acetabular component based on a pelvic AP  Radiograph  Radiographic follow-up was performed at 3 months, 1 year, 2 years, 5 years, and then every 5 years thereafter. | The majority (58% [120 of 206]) of dislocated  THAs had a socket within the Lewinnek safe zone. Mean cup inclination was 44° ± 8° with 84% within the safe zone for inclination. Mean anteversion was 15° ± 9° with 69% within the safe zone for anteversion. Sixty-five percent of dislocated THAs that were performed through a posterior approach had an acetabular component within the combined acetabular safe zones, whereas this was true for only 33% performed through an anterolateral approach. acetabular components performed through a posterior approach (OR, 1.6; 95% CI, 1.2–1.9) had an increased risk of dislocation compared with those performed through an anterolateral approach (OR, 0.8; 95% CI, 0.7–0.9; *P* < 0.0001). The authors found that the historical target values for cup inclination and anteversion defined by Lewinnek et al. may be useful but should not be considered a safe zone, in the sense that positioning the acetabular component within these parameters does not preclude dislocation. | 3 |
| 27 | Furuichi et al., 2016 | To investigated spinopelvic alignment after THA | Retrospective case-control study | 135 patients (13 male and 122 female) with hip OA who underwent  THA surgery; Average age 66 (9.4) years | THA; | Standing APP, SS, and LL  Position: Total spine lateral and pelvic lateral radiograms were obtained in the standing position using plain radiography  Radiographic data were obtained before surgery and at 12 months after surgery | Changes in spinopelvic alignment occurred after THA and improved in certain patients. Patients with PR and reduced LL before THA did not demonstrate any change in spinopelvic alignment after THA. Half of the cases with a favourable spinopelvic alignment before THA maintained this favourable condition after THA as well. However, in cases where LL was decreased despite the presence of a favourable spinopelvic alignment, postoperative PR and worsening of spinopelvic alignment were noted. In cases with preoperative PA, high SS, 41% of patients had improved spinopelvic alignment after THA. | 3 |
| 28 | Tamura et al., 2016 | 1. To investigate the chronological changes of pelvic sagittal inclination during a 10-year follow-up period after total THA 2. To report the characteristics of patients who showed a greater than 10° change in the pelvic sagittal inclination from the supine to the standing position | Retrospective review study | 70 patients  (56 females, 14 males) with THA; Average age 54.9 (10.8) years [range 23–77 years] | THA; | Pelvic sagittal inclination in the supine and standing positions  Position: Anteroposterior radiographs of the pelvis in both the supine and standing positions  Radiographic data were obtained before surgery and at 1, 5, and 10  years after THA | Supine pelvic sagittal inclination showed less than 5° of change, whereas standing pelvic sagittal inclination showed a significant decrease with time over the 10-year period. This pelvic sagittal inclination change did not reach the level that it caused negative consequences such as late dislocation.  Pelvic sagittal inclination in the supine and standing positions at each time point:  **Before THA:**  pelvic sagittal inclination (supine): 5.7 (8.6); pelvic sagittal inclination (standing): 4.4 (10.6); *P* = 0.09  **One year after THA:**  pelvic sagittal inclination (supine): 2.4 (9.6); pelvic sagittal inclination (standing): 0.2 (10.8); *P* < 0.01  **Five years after THA:**  pelvic sagittal inclination (supine): 2.1 (9.2); pelvic sagittal inclination (standing): -0.9 (10.5); *P* < 0.01  **Ten years after THA:**  pelvic sagittal inclination (supine): 2.8 (9.9); pelvic sagittal inclination (standing): -7.0 (14.8); *P* < 0.01  Percentages of patients who showed posterior PT more than 10° from the supine position to the standing position increased during 10 years of follow-up. | 3 |
| 29 | Suzuki et al., 2016 | To assess changes in PT from the preoperative supine position to the standing position at 5 years after THA | NR*  * It seems that this study was a prospective cohort study | 77 patients (64 female, 13 male) with THA; Average age 64.4 years [range 44–82 years] | THA; mini-incision direct  lateral approach | PT  Position: Anteroposterior radiographs of the pelvis were taken in the supine and standing positions  Radiographic data were obtained before surgery and at 1 year after THA | PT in 8% of all patients was ≤ −20°, and the greatest PT was −25°. In patients with THA, posterior PT continued up to 5 years after THA. | 2 |
| 30 | Tripuraneni et al., 2016 | To assess difference in CI and CA between DAA and posterior THA | Retrospective, matched cohort study | 132 patients (52 male, 80 female); Average age 60.2 | THA; direct anterior and posterior | CI, CA  Position: Anteroposterior radiograph of the pelvis and cross table lateral hip radiographs were taken  Radiographic data were obtained at minimum of  3 months postoperatively | No observable difference was seen in CI and CA between the two groups.  **DAA group:**  CI: 43.8°; CA: 28°  **DAA group:**  CI: 41.9°; CA 27.9° | 3 |
| 31 | Weng et al., 2016 | To investigate changes in sagittal spinal–pelvic–leg alignment after THA in patients with severe  hip OA, and whether these changes contribute to LBP relief | Prospective study | 69 patients (25 male, 44 female) with a primary diagnosis of unilateral hip OA who elected to undergo primary THA | THA; lateral modified Hardinge  approach | PI, PT, SS, PFA, LL,  T_1_-Spi, LBP  Position: Standing lateral digital radiographs  Radiographic data were obtained preoperatively and at 3, 6 months, and 1 year postoperatively | **Preoperative:**  SS 39.9° (10.4°), PT 9.5° )8.9°), PI 49.3° (11.1°), LL 50.1° (12.7°), PFA 0.3° (6.3°), T_1_-Spi -0.0° (3.1°)  **Postoperative**  SS 39.2° (8.8°), PT 11.0° (8.6°), PI 49.1° (8.9°), LL 50.4° (11.2°), PFA 5.4° (5.1°), T_1_-Spi -2.1° (2.6°)  LBP was reported by 39 patients (56.5 %) before surgery; at follow-up, 17 reported complete resolution, while 22 reported significant relief. | 2 |
| 32 | Keshmiri et al., 2015 | The purpose of this investigation was to  compare clinical outcome between ‘‘cup first’’ navigated and conventional cementless THA 5–7 years after surgery | Retrospective matched-pair study | 50 patients (28 female, 22 male) who had received primary unilateral THA due to primary OA; Average age 63.05 (9.05) years | THA with or without the use of an imageless  navigation system (Hip unlimited 5.0; BrainLAB AG, Feldkirchen, Germany); minimally invasive single incision anterolateral approach | CI  Position: Anteroposterior radiographs  Radiographic data were obtained at a mean of 6.4 (4.8–7.4) years postoperatively | CI was more closely to the target value of 45° within the navigated THA group with a mean cup inclination of 45.3° (SD 4.2°; range 40.0°–58.0°), while the conventional THA group showed a mean inclination of 48.0° (SD 4.2°; range 41.4°–59.4°) (*P* = 0.02). | 3 |
| 33 | Maratt et al., 2015 | To assess the incidence of standing PT in patients undergoing primary unilateral THA | Cohort study | 138 patients (67 female, 71 male) who underwent unilateral primary THA; Average age 57 (11) years | THA; | PT, PI, APP, CA, FI  Position: Standing lateral radiographs  Radiographic data were obtained preoperatively and 6 weeks postoperatively | Mean preoperative PT was 0.6° (SD 7.3°; range: −19.0° to 17.9°). Mean postoperative PT was 0.3° (SD 7.4°; range: −18.4° to 15.0°). Mean change in PT was −0.3° (SD 3.6°; range: −9.6° to 13.5°), with no significant difference between preoperative and postoperative PT (*P* = 0.395). Preoperative PT was strongly correlated with postoperative PT (*r^2^* = 0.88, *P* = 0.0001). PI did not correlate with change in PT (*r^2^* = −0.16, *P* = 0.06). The FI of the acetabular component showed a nonlinear response to change in PT. This was especially apparent in combination with high APP anteversion. | 3 |
| 34 | Tezuka, 2014 | To investigate changes in PIA between the supine position and standing position after THA, and the correlation between cup position and PIA | NR | 53 patients who underwent primary cementless THA; Average age 59 (6.9) years | THA; | PIA, CI, and CA in the supine and standing positions | CI was 40.2° (6.7⁰) in the supine position and 41.9° (7.1⁰) in the standing position (*P* < 0.01), while CA was 14.5° (5.5⁰) in the supine position and 18.2° (6.3⁰) in the standing position (*P* < 0.01). Furthermore, PIA was 24.7⁰ (7.5⁰) in the supine position and 31.6⁰ (9.7⁰) in the standing position (*P* < 0.01). | 3 |
| 35 | Radcliff et al., 2013 | To assess the difference in sagittal plane lumbar alignment between patients with THA and patients with lumbar DDD | Prospective study | 12 patients with THA (average age 64.2 years) and 34 patients with DDD (average age 63 years) | THA; anterolateral approach. | SS, LL, L_1_ axis to S_1_ distance  Position: Upright, standing, lateral radiographs  Radiographic data were obtained preoperatively and approximately 9 months postoperatively | The sacral slope was greater in the THA group  (mean, 23.4°) than in the lumbar DDD patients and this difference was statistically significant (mean, 11.6°, *P* = 0.001). There were no statistically significant differences between THA and lumbar DDD patients in lumbar lordosis (46.1° vs. 49.9°; *P* >0.05) or L1 axis to S1 distance (35.5° vs. 35.6°; *P* >0.05). Change before and after THA was measured. There were no statistically significant differences in assessed radiographic variables preoperatively versus postoperatively. | 2 |
| 36 | Murphy et al., 2013 | To assess variation in PT in patients before THA and the effect of THA on PT in the same patients more than a year after THA | Prospective study | 30 patients (15 female, 15 male); Average age 59.9 (9) years (range 37-80 years) | THA; | PT  Position: Standing and supine anteroposterior pelvic radiograph; and CT scan  Radiographic data were obtained before and after surgery | **Preoperative supine PT:**  Mean: 4.4°, SD: 6.4°, range: -7.7° – +20.8°  **Preoperative standing PT:**  Mean: 1.5°, SD: 7.2°, range: -13.1° – +12.8°  **Postoperative supine PT:**  Mean: 6.3°, SD: 6.6°, range: -5.7° – +19.6°  **Postoperative standing PT:**  Mean: 2°, SD: 8.3°, range: -12.3° – +16.8°  **Change in supine PT:**  Mean: 1.9°, SD: 3.3°, range: -8.5° – +5.5°  **Change in standing PT:**  Mean: 0.5°, SD: 3°, range: -5° – +7.15°  Preoperative PT correlated with postoperative PT in both the standing (*r^2^* = 0.87) and supine (*r^2^* = 0.75) positions. | 2 |
| 37 | Polkowski et al., 2012 | To assess whether standing changes standard measurements of acetabular component position using a novel biplanar imaging system compared  to traditional supine imaging | Prospective study | 23 patients with THA | THA; | Sagittal plane anteversion  Position: Supine for conventional radiograph and standing for EOS^®^ radiograph  Radiographic data were obtained after surgery | Standing anteversion differed from supine anteversion by greater than 5° for 12 of 23 patients who underwent THA (range, 5°–16°). | 2 |
| 38 | Taki et al., 2012 | To evaluate the change in PT in the  sagittal plane in the standing and supine positions for 1 to 4 years after THA | Retrospective study | 86 patients (65 female, 21 male); Average age 64 [range 39-85] years | THA; | PT  Position: Standardized anteroposterior radiographs of the pelvis in the supine and standing positions  Radiographic data were taken before, 1 year, and 2-4 years after surgery | **Before THA (*n* = 86):**  Standing PT: 22.6° (10.4°); Supine PT: 18.5° (8°)  **1 year after THA (*n* = 86):**  Standing PT: 26.5° (10.5°); Supine PT: 21.2° (7.3°)  **2 years after THA (*n* = 86):**  Standing PT: 27.9° (10.6°); Supine PT: 21.1° (7.1°)  **3 years after THA (*n* = 55):**  Standing PT: 26.7° (10.9°); Supine PT: 20.8° (7.8°)  **4 years after THA (*n* = 55):**  Standing PT: 29.1° (11.7°); Supine PT: 22.3° (7.4°)  The percentage of patients who showed a difference of more than 10° in change in PT tended to increase yearly. In addition, the older patients showed a larger change in PT after THA. | 3 |
| 39 | Lazennec et al., 2011 | To compare measured acetabular cup orientations on axial CT scans taken with the patient in a  supine position with those on CT sections at angles to the  sacral slope reflecting standing and sitting positions | Retrospective study | 328 asymptomatic patients (157 male, 171 female) with THA | THA; | SS, CI, CA  Position: anteroposterior and lateral radiographs of  the pelvis in the standing and sitting positions and a CT scan of the pelvis in a supine position | **SS:** supine 45.6°±9.8°; standing 35.0°±10.7°; sitting 20.5°±11.5°  **Acetabular frontal inclination:** supine 46.3°±3.6°; standing 49.4°±4.0°; sitting 56.7°±7.1°  **Acetabular sagittal inclination:** supine 25.5°±8.4°; standing 36.0°±8.2°; sitting 50.6°±11.2°  **Anatomic acetabular anteversion:** supine 24.2°±6.9°; standing 31.7°±5.6°; sitting 38.8°±5.4°. | 3 |
| 40 | Ishida et al., 2011 | 1. To assess changes in PT after THA. 2. To assess the correlation between preoperative PT and the extent of postoperative changes in the PT | Prospective study | 149 patients  (28 male, 121 female) who underwent primary THA; Average age 64 years [range 38-83) | THA; anterolateral  approach | PT  Position: anteroposterior radiographs of the pelvis in lying and standing positions, and lateral radiographs of the pelvis in the standing position  Radiographic data were obtained before, 3 and 6 months and 1 year after surgery | A significant correlation was reported between preoperative APP and the amount of postoperative change in APP, suggesting that patients with severe preoperative PT generally experience greater postoperative changes in PT. | 2 |
| 41 | Zhu et al., 2010 | To determine the distribution and magnitude of PT, especially tilt of 10⁰ or greater, in patients undergoing THA in the lateral decubitus position | Prospective study | 436 patients (477 hips; 216 male, 220 female) who underwent primary THA; Average age 62.9 years [range 23-89] years | THA; | PT  Position: the patient was positioned in the side bending position  Radiographic data were taken after surgery | The distribution of PT had a range of 25° posterior to 20° anterior. Twenty-nine of 477 (6.1%) hips had zero tilt; 251 (52.6%) had PT of 1° to 5°; 120 (25.2%) had tilt of 6° to 9°; and 77 (16.1%) had tilt of ≥ 10°. | 2 |
| 42 | Parvizi et al., 2010 | 1. To identify the incidence/prevalence of LBP in a group of patients with end-stage arthritis of the hip undergoing THA 2. To describe the natural history of LBP in this cohort undergoing THA 3. To determine factors that were predictive of persistent LBP after THA | Prospective study | 344 patients (165 male, 179 female) who underwent primary THA; Average age 64.5 years [range 32.8–87.1] years | THA; | LBP | Postoperatively, the LBP resolved in 113 (66.4%) of the 170 patients. Thirty-seven of the remaining 57 patients had known spine disorders. Thirty-five of the 174 patients (20%) without prior LBP had LBP develop within 1 year postoperatively. The LBP improved in 17 of these 35 patients; 12 of the remaining 18 patients had preexistent spine disorders. Pain radiating below the knee was associated most closely with preexisting spine disorders | 2 |
| 43 | Blondel et al., 2009 | To evaluate the reproducibility of PT measurement  and its variability between THA preoperative and 3-year postoperative measurements | Prospective study | 50 patients (26 male, 24 female); Average age 64 years [range 47-81 years] | THA; Watson-Jones anterolateral approach | PT, PI  Position: Lateral radiographs were obtained in the standing position with the hip in extension and the arm resting at 90⁰ on a support, at equal distance from the tube, on large radiographic tapes  Radiographic data were obtained before and 3 years after surgery | There was no statistically significant variation between preoperative and 3-year follow-up values (*P* > 0.05). Ninety-five percent of the patients had less than a 5° difference between both measurements while 5% had a difference ranging from 5° to 10°; none of the patients reported a variation greater than 10°.  Preoperative PT: Mean: 4.68° (range -6° to 14°)  Preoperative PI: Mean: 56.04° (range 40° to 87°)  Postoperative PT: Mean: 4.78° (range -5° to 14°)  Postoperative PI: Mean: 55.96° (range 40° to 83°) | 2 |
| 44 | DiGioia et al., 2006 | 1. To assess pelvic flexion extension between different patients in the same position and different positions for the same patient 2. To compare preoperative and postoperative pelvic flexion extension | Prospective study | 84 patients (44 male, 40 female); Average age 62 years [range 37-81 years] | THA; | Pelvic orientation (flexion or extension), pelvic motion arc (flexion extension)  Position: lateral radiograph  Radiographic data were obtained before and 3 months after surgery | There was approximately upright pelvic alignment during standing, with a mean APP angle of 1.2° (range −22° to +27°). In the sitting position the pelvis tended to extend posteriorly, with a mean APP angle of −36.2° (range −64° to +4°). There was a large variation in the arc of pelvic motion when patients  moved from standing to sitting positions, with arc of pelvic motion ranged from 5° to 70°. No significant sex-related differences were found and there were no significant differences between preoperative and postoperative pelvic motion arc as well. | 2 |
| 45 | Nishihara et al., 2003 | To evaluate  whether safe acetabular component position depends on differences in pelvic location between  the supine, standing, and sitting positions | NR*  * It seems that this study was a prospective cohort study | 101 patients (30 male, 71 female) who had primary or revision THA; average age 56 years [range 23-81 years] | THA; | PT  Position: Anteroposterior radiographs of the pelvis in the supine, standing, and sitting positions; and CT scan  Radiographic data were obtained before and 1 year after surgery | The mean preoperative anterior PT was 5° to 9° (range -37° to 30°) in the supine position, 3° to 12° (range -46° to 33°) in the standing position, and -29° to 12° (range -62° to 10°) in the sitting position. In 90% of the cases, the difference in anterior PT between the supine and standing positions preoperatively was ≤ 10°. In 90% of the cases, there was ≥ 20° extension of the pelvis from the supine position to the sitting position preoperatively. Preoperative pelvic position in each case was almost completely maintained 1 year following THA. | 2 |
| **Abbreviations:** APP: sagittal anterior pelvic plane; CA: cup anteversion; CI: cup inclination; CL: cervical lordosis; CSI: combined sagittal index; CT: computerized tomography; DAA: direct anterior approach; DDD: degenerative disc disease; DDH: developmental dysplasia of the hip; FCA: functional cup anteversion; FI: functional inclination; HOA: hip osteoarthritis; IQR: interquartile range; LBP: low back pain; LL: lumbar lordosis; LSF: lumbar spine fusion; NHD: neglected hip dysplasia; NR: not reported; ODI: Oswestry disability index; OR: odds ratio; PA: pelvic anteversion; PFA: pelvic femoral angle; PI: pelvic incidence; PIA: pelvic inclination angle; PR: pelvic retroversion; PT: pelvic tilt; SS: sacral slope; SVA: sagittal vertical axis; T_1_Spi: T_1_-spinopelvic inclination; THA: total hip arthroplasty; TK: thoracic kyphosis; TPA: T_1_ pelvic angle; VAS: visual analogue scale. | | | | | | | | |
